# Real‐Time Biomass Estimation in High‐Density Yeast Fermentations Using Soft Sensor Modeling

**DOI:** 10.1002/bit.70266

**Published:** 2026-06-11

**Authors:** Ana G. Del Hierro, José Luis Checa‐Barrera, Juan A. Moreno‐Cid, Eoin Casey

**Affiliations:** ^1^ School of Chemical and Bioprocess Engineering University College Dublin, Belfield Dublin Ireland; ^2^ BiOrbic, Research Ireland Centre for Bioeconomy Dublin Ireland; ^3^ Bioprocessing R&D Department, Bionet, Parque Tecnológico Fuente Álamo Fuente Álamo Spain; ^4^ R&D Department Probelte SAU – Agronova Biotech Molina de Segura Spain

**Keywords:** biomass estimation, bioprocess monitoring, cross‐validation, optical density, regression‐modeling, soft sensor, yeast fermentation

## Abstract

Accurate biomass monitoring remains a bottleneck in industrial fermentation, where conventional offline methods, gravimetric dry cell weight (DCW) and spectrophotometric optical density at 600 nm (OD_600_), are incompatible with real‐time process control. Online optical probes operating at 860 nm (OD_860_) provide continuous, non‐invasive monitoring, but their signals become increasingly nonlinear due to multiple light scattering, limiting direct interpretation without mathematical transformation. This study develops and validates interpretable soft sensor regression models that convert nonlinear OD_860_ signals into DCW and OD_600_ across three industrially relevant yeast species, *Pichia pastoris*, *Kluyveromyces marxianus*, and *Yarrowia lipolytica*. Fed‐batch datasets from 19 independent batches were used to evaluate 14 candidate models, including nonlinear and spline‐based functions. Model performance was assessed by the nested leave‐one‐batch‐out cross‐validation (LOBO‐CV) to prevent information leakage from batch‐correlated data. Model selection followed a parsimony criterion, retaining the simplest model within 5% of the minimum cross‐validated RMSE. For DCW prediction, selected models achieved cross‐validated coefficients of determination (*R* ^2^) of 0.85–0.91 across species; OD_600_ predictions were less accurate (*R* ^2^_CV_ = 0.49*–*0.90), reflecting dilution‐driven noise amplification inherent to high‐density spectrophotometric measurements. A dual‐model strategy is presented, pairing software‐optimized implementations with simplified piecewise‐linear equations suitable for calculator‐based deployment in industrial environments.

## Graphical abstract

Accurate biomass measurements, both offline and online, are essential to improve prediction and control in yeast fermentation. This study develops regression‐based predictive models to correlate offline measurements (Dry Cell Weight and OD_600_ from a spectrophotometer) with online OD_860_ probe signals to provide accurate real‐time biomass estimations and improve process understanding and control.

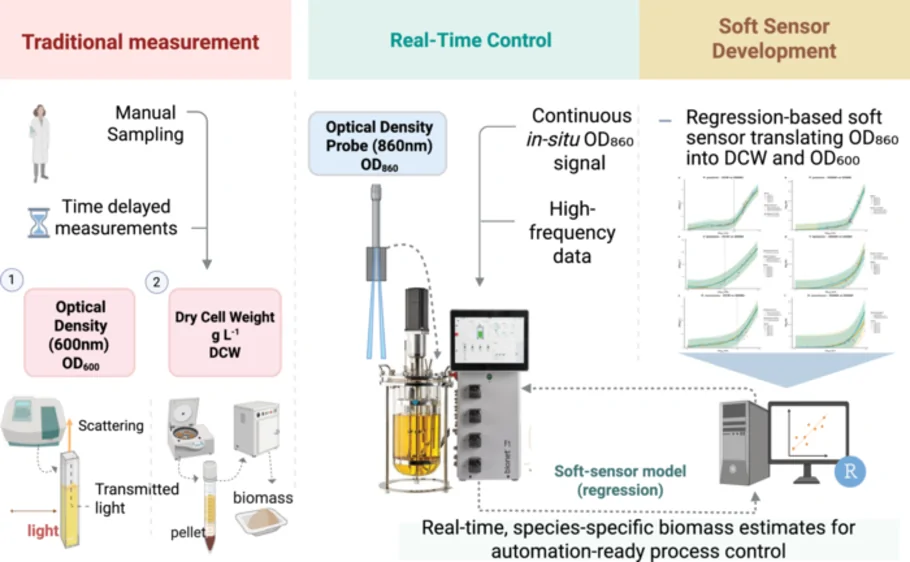

**Abbreviations**
ANCOVA: analysis of covariance
AU: arbitrary units
BP: breakpoint
BS: basic spline
CECT: Spanish‐Type Culture Collection
CI: confidence interval
CI_95_
: 95% confidence interval
CIP: clean‐in‐place
CRL1: *Candida rugosa* Lipase 1
CV: coefficient of variation
DCW: dry cell weight
EDF: effective degrees of freedom
FIJI: Fiji Is Just ImageJ
FOV: field of view
GAP: glyceraldehyde‐3‐phosphate dehydrogenase
KOH: potassium hydroxide
LOBO‐CV: leave‐one‐batch‐out cross‐validation
LOGO‐CV: leave‐one‐group‐out cross‐validation
LSTM: long short‐term memory
MCYC: Microbial Culture Collection Yeast
NCYC: National Collection of Yeast Cultures
NH_4_OH: ammonium hydroxide
NIR: near‐infrared
NS: natural spline
OD: optical density
OD_600_
: optical density at 600 nm
OD_860_
: optical density at 860 nm
PAT: process analytical technology
PM: production medium
*R* ^2^
: coefficient of determination
*R* ^2^_CV_
: cross‐validated coefficient of determination
*R* ^2^ REFIT: Refit model coefficient of determination
RMSE: root mean square error
RMSE_CV_
: cross‐validated root mean square error
RMSEREFIT: Refit model root mean square error
RNN: recurrent neural network
RPM: revolutions per minute
SD_cv_
: standard deviation of cross‐validation
TES: trace element solution
UAB: Universidad Autónoma de Barcelona
VVM: volume per volume per minute

## 1 Introduction

Accurate biomass monitoring is fundamental to maintaining optimal conditions and productivity in industrial fermentations (Kiviharju et al. 2007; Schiewe et al. 2023). Bioprocess performance depends on reliable process indicators obtained through online, at‐line, and offline measurements (Biechele et al. 2015; Kiviharju et al. 2008; Schiewe et al. 2023; Vaidyanathan et al. 1999). Gravimetric dry cell weight (DCW) remains the reference standard for biomass quantification; yet its requirement for centrifugation and drying precludes real‐time application and renders it incompatible with closed‐loop process control (Bauer et al. 2022; Brunner et al. 2020; Kiviharju et al. 2007). Optical density at 600 nm (OD_600_), measured with a standard bench‐top spectrophotometer, is widely used as a biomass proxy owing to its operational simplicity. However, it requires serial dilution at high cell densities, where multiple light‐scattering effects cause the measured signal to diverge progressively from true biomass concentration (Myers et al. 2013; Ulber et al. 2003).

**Figure 1:**
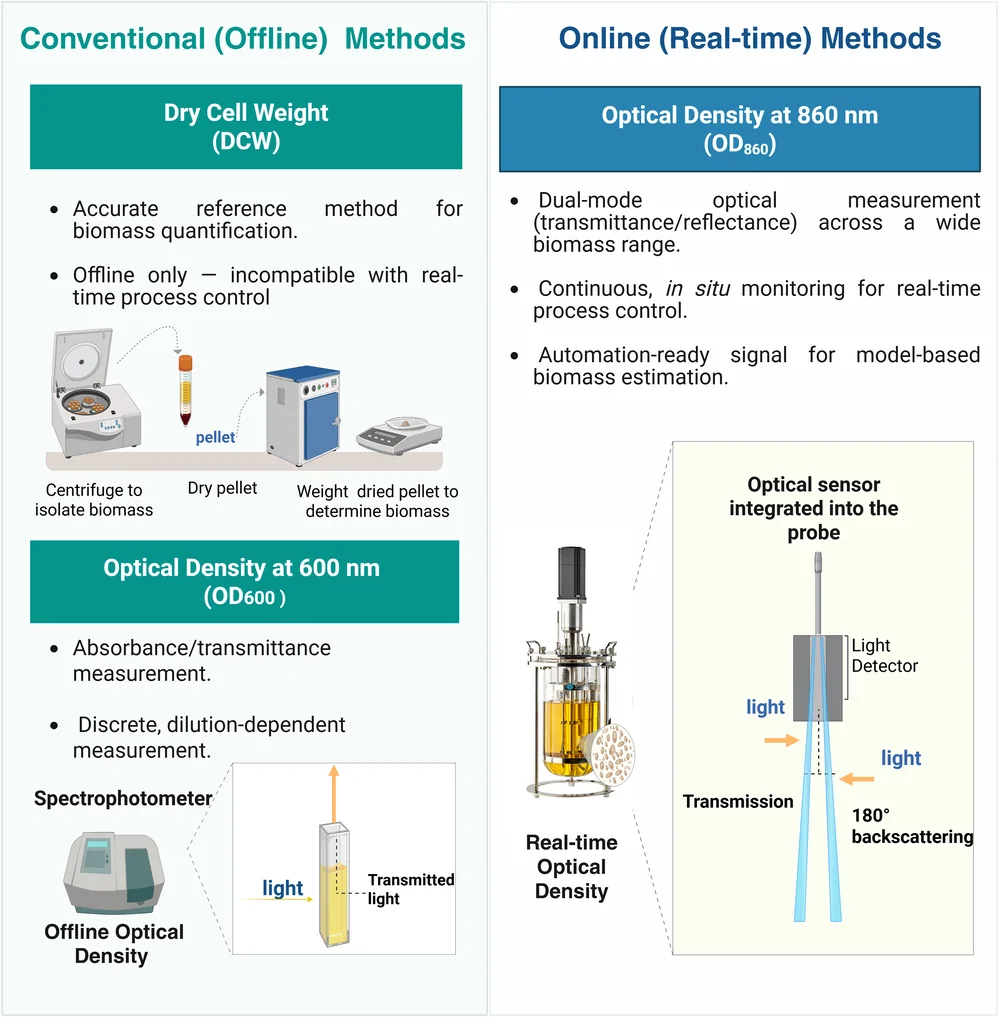
Conventional offline methods, gravimetric dry cell weight (DCW) and spectrophotometric optical density at 600 nm (OD_600_), serve as reference measurements for biomass quantification but require manual sampling, centrifugation, and serial dilution, precluding real‐time application. DCW is obtained by centrifugation, pellet drying, and gravimetric measurement, and constitutes the primary calibration reference in this study. OD_600_ is measured in a cuvette‐based spectrophotometer; at high cell densities, light scattering causes progressive deviation from Beer–Lambert linearity, necessitating dilution. In contrast, the Online OD_860_ probe (Hamilton Dencytee) measures transmission and reflection optical currents simultaneously and combines them through an internal manufacturer‐calibrated correlation to produce a single OD_860_ signal in absorbance units, extending the measurable biomass range continuously and without sample manipulation. The offline DCW and OD_600_ measurements serve as calibration targets for the soft sensor models developed from the Online OD_860_ signal.

To enable continuous, non‐invasive monitoring, single‐wavelength near infrared (NIR) probes operating in the 840–910 nm range have been developed for direct in situ turbidity measurement, a window in which media components exhibit minimal absorption (Biechele et al. 2015; Kiviharju et al. 2008). At high biomass concentrations, however, multiple‐scattering effects distort the relationship between the online OD_860_ signal and true biomass, making online vs. offline direct conversion unreliable without appropriate calibration (Cervera et al. 2009; Myers et al. 2013; Ulber et al. 2003). The operational contrast between offline reference methods (DCW, OD_600_) and the online OD_860_ signal is illustrated in Figure 1.

Soft sensors, computational models that infer unmeasured process variables from correlated online measurements, have consequently emerged as a practical route to translating online readings into actionable biomass estimates in real time (Luttmann et al. 2012). Existing soft sensor strategies for biomass estimation fall into four broad categories. Balance‐based and hybrid models couple off‐gas analysis with stoichiometric or dynamic equations to infer biomass from respiratory patterns (Brunner et al. 2020; Jenzsch et al. 2006; Sagmeister et al. 2013). These achieve strong predictive accuracy but require dedicated gas‐analysis hardware and validated mass‐balance closure. Multivariate spectroscopic approaches exploit broadband NIR signals with chemometric calibration (Biechele et al. 2015; Cervera et al. 2009; Ulber et al. 2003), providing rich spectral information at the cost of complex preprocessing and probe‐dependent recalibration. Deep‐learning architectures, notably long short‐term memory (LSTM) recurrent neural network (RNN) have been applied to *Pichia pastoris* fermentations, which accommodate inter‐batch distributional shift but reduce model interpretability (Wang et al. 2025). Hardware‐based solutions such as Photon Density Wave spectroscopy physically circumvent optical nonlinearity but require instrumentation absent from standard bioprocess facilities (Schiewe et al. 2023). Single‐wavelength in situ NIR probes are already applied across a large fraction of industrial bioreactors and represent the most accessible route to online biomass monitoring. Yet their calibration has historically been confined to linear or low‐order polynomial fits within individual species (Kiviharju et al. 2008).

The present study develops and validates soft sensor regression models for real‐time biomass estimation in high‐cell‐density fed‐batch fermentations across three physiologically distinct industrial yeast species: recombinant *Pichia pastoris* (GAP promoter; pGAP), wild type *Kluyveromyces marxianus* (NCYC 152), and wild type *Yarrowia lipolytica* (MCYC 2060). The online OD_860_ signal is the sole process input, avoiding dependence on off‐gas analyzers or multivariate spectroscopic systems. Species‐specific breakpoints were identified using segmented regression (Muggeo 2003) to separate quasi‐linear and nonlinear scattering regimes within each dataset. Fourteen candidate models, including nonlinear and spline‐based functions, were then evaluated systematically. Model selection followed a parsimony criterion, retaining the simplest model within 5% of the minimum cross‐validated RMSE to limit overfitting. Performance was assessed across species using nested leave‐one‐batch‐out cross‐validation (Cawley and Talbot 2010), establishing the transferability boundaries of single‐wavelength calibration across yeasts. The resulting models translate OD_860_ signals into conventional biomass indicators, DCW and OD_600_, improving the interpretability and practical usability of online optical monitoring.

## 2 Materials and Methods

### 2.1 Strains and Media Preparation

Unless otherwise stated, all reagents used in this work were purchased from Sigma‐Aldrich (Darmstadt, Germany). Media formulations for each yeast strain were prepared as detailed below. The strain of *P. pastoris (syn. Komagataella phaffii)* with the pGAP promoter to express *Candida rugosa lipase 1 (CRL1*) was provided by the Bioprocess Engineering and Applied Biocatalysis Group of the Universidad Autónoma de Barcelona (UAB, Barcelona, Spain). *Y. lipolytica* (MCYC 2060) and *K. marxianus* (NCYC 152) strains were acquired from the Spanish‐type culture collection (CECT).

Production medium (PM) for *P. pastoris*, *K. marxianus*, and *Y. lipolytica* was prepared following established protocols detailed in (Gasset et al. 2022; Lukondeh et al. 2005; Xie et al. 2017), respectively. Each yeast was grown in a batch followed by a nutrient‐limited fed‐batch phase. Trace Element Solution (TES) and vitamins were filter‐sterilized and aseptically added to the production and fed‐batch media post‐autoclaving to maintain stability and effectiveness.

### 2.2 Bioreactor Operation

Each strain was cultivated in a 3 L benchtop microbial bioreactor (model F1‐3MB, Bionet, Spain). The PM and the bioreactor were sterilized at 121°C for 30 min. During sterilization, an antifoam agent (ACEPOL 69, Dystar, Singapore) and pH control solutions were autoclaved along with the PM. Potassium hydroxide (KOH) was employed to adjust the pH to 5.5 ± 0.05 for *K. marxianus* and *Y. lipolytica*. In contrast, ammonium hydroxide (NH_4_OH), which does not require sterilization, was added to the *P. pastoris* reactor post‐sterilization for pH control.

The cultivation began with a working volume of 1.6 L of the respective PM. Bioreactor parameters were managed using ROSITA software (Bionet, Spain). Dissolved oxygen (pO_2_) levels were maintained at 30% air saturation through cascade agitation (500–1800 revolutions per minute, rpm) and pure oxygen enrichment. The total gas flow rate was set at 1.5 volume of gas per volume of liquid per minute (vvm). Temperature was controlled at 25°C ± 0.1°C for *P. pastoris* and 30°C ± 0.1°C for *Y. lipolytica and K. marxianus*.

Each yeast strain was grown in a batch followed by a nutrient‐limited fed‐batch phase at a defined specific growth rate (*µ*). *P. pastoris* was cultured for 18 h in batch before a fed‐batch phase at *µ* = 0.05 h^−1^. *Y. lipolytica* was grown for 7 h in batch, followed by a fed‐batch at *µ* = 0.2 h^−1^. *K. marxianus* was also cultivated for 7 h in batch and then fed at a rate of *µ* = 0.5 h^−1^.

### 2.3 Online Biomass Monitoring

The OD_860_ sensor Dencytee RS485 325 Arc Sensor (Hamilton, Bonaduz, Switzerland; part no. 10064919‐12, optical path length 5 mm, *λ* = 860 nm) was calibrated to zero in sterile media before inoculation. Data were acquired at 1‐min intervals using ROSITA software (Bionet, Spain). To isolate potential electronic drift or biofilm interference from cleaning artefacts, sensor stability was verified post‐harvest but prior to any clean‐in‐place (CIP) procedure. In all runs, the OD_860_ signal returned to the initial baseline with < 3% variation. Final visual inspections confirmed the absence of optical window fouling, ensuring measurement integrity throughout each fermentation.

#### 2.3.1 Brightfield Microscopy and Cell Morphometry

To characterize cell morphology and verify the Mie scattering regime assumptions underlying the optical signal interpretation, brightfield microscopy was performed in one additional batch conducted for morphological analysis, separately from the 19 batches employed for soft sensor model training and validation. For each species, five samples were collected across the optical range observed during modeling, including points above the OD_860_ breakpoint where multiple‐scattering effects become dominant spanning a comparable density range (Low, Mid‐low, Mid, Mid‐high, High bands).

Brightfield images were recorded using an HR‐5000E microscope (HR High‐Range lens, 3000×, Hirox Co. Ltd.; FOV = 102.00 µm, 2000 × 1500 pixels, 0.051 µm·px^−1^). Scale calibration was applied in FIJI (ImageJ v1.54, NIH, USA) (Rueden et al. 2017; Schindelin et al. 2012) using the instrument‐embedded scale bar (20 µm = 392 pixels). For each image, well‐defined non‐overlapping cells were segmented using the FIJI Analyze Particles function with ellipse fitting enabled and Otsu thresholding (Otsu 1979). The Feret diameter (maximum caliper distance, µm) was adopted as the primary morphological descriptor, providing a robust geometric size metric for the Mie size parameter *x* = πd/λ for non‐spherical particles randomly oriented in suspension (Bohren and Huffman 1983; van de Hulst 1957). The median Feret diameter and the cell‐by‐cell coefficient of variation (CV, %) were computed per sample.

### 2.4 Offline Biomass Monitoring

Offline OD_600_ biomass sampling was performed every 2 h during both batch and fed‐batch phases to provide reference data for model training and validation. OD_600_ was measured using a Zuzi 4111 R spectrophotometer (Daselab, Spain), applying serial dilutions (ranging from 1:10 to up to 1:1000 at the highest concentrations) to maintain absorbance values within the linear range (< 0.9).

Dry cell weight (DCW) was determined gravimetrically by centrifuging 20 mL of culture in pre‐weighed centrifuge tubes using a Digicen 21 centrifuge (Orto Alresa, Madrid, Spain) equipped with an RT 154 angle fixed rotor. Samples were centrifuged at 9000 rpm (9146*g*) for 20 min. The supernatant was discarded, and the pellets were washed once with deionised water. Tubes containing biomass were dried at 80°C for 24 h to prevent thermal degradation and volatilization of unsaturated fatty acids in oleaginous biomass such as *Y. lipolytica*, then cooled to room temperature on the bench and then weighed (Papanikolaou et al. 2001, 2002). The same protocol was applied identically to all three species to ensure an internally consistent gravimetric reference. DCW (g·L^−1^) was calculated as the mass difference between the dried tube and the initial tube mass divided by the sample volume (Kaushik et al. 2020; Papanikolaou et al. 2001).

### 2.5 Data Preparation

Across the three strains, this procedure yielded a total of 173 paired online‐offline data points distributed across nineteen independent batches (five batches for *P. pastoris*, seven for *Y. lipolytica*, and seven for *K. marxianus*), ensuring consistent temporal resolution for regression model development (Supporting Information S1: IA). Data were screened for integrity and outliers. Given the limited number of independent batches per strain (5–7), nested leave‐one‐batch‐out cross‐validation (LOBO‐CV), equivalent to leave‐one‐group‐out cross‐validation (LOGO‐CV) in machine learning literature, was selected for model evaluation to maximize data utilization while respecting the hierarchical batch structure and preventing information leakage from temporally correlated observations (Arlot and Celisse 2010; Varma and Simon 2006). No normalization or scaling was applied, as all variables were measured on comparable numerical scales, ensuring direct interpretability of regression coefficients.

### 2.6 Model Development

The predictive model was designed around a single input variable (OD_860_) to capture the relationship between the continuous optical response and conventional biomass indicators. This single‐predictor approach reflects both the physical measurement principle (a single optical wavelength) and the limited dataset size, which increases the risk of overfitting with multiple predictors. A comprehensive model library of 14 regression approaches was established, encompassing parametric and semi‐parametric methods (Table 1).

**Table 1:** Mathematical models evaluated for describing the relationship between biomass concentration and optical signal OD860. Model Class: Categorized into Global Polynomials (Poly), Exponential, Power‐law (Freundlich), and Splines (Piecewise B‐splines and Natural Splines). Formula: The functional form of the model; y represents the optical response, while a, b, c, d and e are fitting parameters. For Spline models, EDF quantifies model complexity and accounts for the number of basic functions used to construct the piecewise curve higher EDF increases both flexibility and overfitting risk.

Model	Formula	EDF	Rationale for Inclusion	Reference
Poly_1	y=a+bx	2	Null model; Beer–Lambert linearity assumption at low cell densities	(Mayerhöfer et al. (2020))
Poly_2	$y=a+\mathrm{bx}+cx^{2}$	3	Quadratic saturation capturing signal deceleration at high OD_860_	(Montgomery et al. (2012))
Poly_3	$y=a+\mathrm{bx}+cx^{2}+dx^{3}$	4	Inflection point detection for sigmoidal optical transitions.	
Poly_4	$y=a+\mathrm{bx}+cx^{2}+dx^{3}+ex^{4}$	5	Higher‐order flexibility for complex nonlinearities.	
Exponential	y = ae^(bx)^	2	Multiple scattering amplification at high densities	
Freundlich	$y=ax^{b}$	2	Power‐law is common in scattering phenomena.	(Freundlich (1907))
Bs_1	Piecewise linear	3	Abrupt optical transition; interpretable breakpoint for operators.	(Hastie et al. (2009))
Bs_2	Piecewise quadratic	4	Smoother transition with continuous first derivative at BP.	
Bs_3	Piecewise cubic	5	Standard B‐spline; continuous first and second derivatives	
Bs_4	Piecewise quartic	6	Maximum local flexibility around breakpoint region	
Ns_2_Bp	Natural spline (knot_BP)	3	Boundary‐constrained fit with knot at segmented regression BP.	(Hastie and Tibshirani (1990))
Ns_2	Natural spline (df=2)	3	Data‐driven knot at median; automatic adaptation.	
Ns_3	Natural spline (df=3)	4	Two internal knots; balance flexibility vs. stability.	
Ns_4	Natural spline (df=4)	5	Three internal knots; maximum flexibility with boundary constraints.	

Abbreviations: BP = breakpoint; EDF = effective degrees of freedom.

Parametric methods included polynomial regression (degrees 1–4), exponential regression (*y* = ae^(bx)^), and the Freundlich power‐law regression ($y=ax^{b}$). Semi‐parametric approaches included basic splines (bs) with polynomial degrees 1–4 and a single breakpoint, and natural splines (ns) with 2–4 degrees of freedom. Model complexity was quantified using effective degrees of freedom (EDF), defined as the number of free parameters in each model (Hastie and Tibshirani 1990). For polynomial and parametric models, EDF equals the number of fitted coefficients, including the intercept. For regression splines, EDF equals the number of basic functions plus intercept: natural splines with df degrees of freedom have EDF=df+1; basic splines with degree d and one internal knot have EDF=d+2. The breakpoint for basic splines was identified using segmented regression (Muggeo 2003), which detects statistically significant shifts in linear relationships.

Critically, breakpoint detection was performed independently within each cross‐validation fold using only training data to prevent information leakage. Model fitting and validation were conducted in R (version ≥ 4.3.0; R Foundation for Statistical Computing, Vienna, Austria) using the splines package for spline construction. Three nonlinear least squares models, Langmuir $(y=\;\frac{\mathrm{ax}}{b+x}$), modified Langmuir $(y=\;\frac{ax^{2}}{b+x^{2}}$), and inverse polynomial $(y=\;\frac{1}{a+\mathrm{bx}}$) were initially included but excluded due to convergence failures in > 70% of cross‐validation folds (Hastie et al. 2009). These failures occurred primarily at low OD_860_ values, where the constrained parameter space prevented stable optimization.

#### 2.6.1 Model Performance

Model performance was assessed using nested leave‐one‐batch‐out cross‐validation (nested LOBO‐CV). In each iteration, one complete batch was held out for validation while remaining batches constituted the training set, yielding *k* = 5–7 fold‐specific performance estimates per strain (*k* = 5 for *P. pastoris*, *k* = 7 for *Y. lipolytica* and *K. marxianus*). The term “nested” refers specifically to the re‐estimation of the optical breakpoint, the OD_860_ threshold separating quasi‐linear from nonlinear scattering regimes—within each cross‐validation fold using only training data. This creates a two‐level structure: an outer loop that iterates over held‐out batches, and an inner estimation step that recalculates the breakpoint via segmented regression (Muggeo 2003) from the training batches of each fold. Because the breakpoint is a data‐dependent preprocessing parameter (not a fixed constant), its estimation from the complete data set would constitute information leakage from the held‐out batch, producing optimistically biased performance metrics (Cawley and Talbot 2010). By re‐estimating the breakpoint within each fold, the cross‐validated metrics (RMSE_CV_, *R* ^2^_CV_) reflect the true predictive performance of the complete modeling pipeline, including breakpoint detection, when applied to an unseen batch. The resulting fold‐to‐fold breakpoint variability (CV < 4% for *P. pastoris* and *K. marxianus*, 15%–20% for *Y. lipolytica*; Supporting Information S1: IB Table S1) provides an additional measure of optical consistency across batches. This design is distinct from classical nested cross‐validation for hyperparameter tuning (Varma and Simon 2006), which employs a full inner k‐fold loop; here, the inner step is a single deterministic estimation rather than an optimization search.

All 14 candidate models were then fitted to the training data, and predictions were generated for the held‐out batch. The primary performance metric was root mean square error (RMSE), $\mathrm{RMSE}=\sqrt{\left[\frac{\sum {\left(y_{i}-{ŷ}_{i}\;\right)}^{2}}{n}\right]}$. RMSE was selected for its sensitivity to outliers, enabling direct comparison with prior soft sensor work (Brunner et al. 2020; Kadlec et al. 2009) and interpretability in original units (g L^−1^ for DCW, dimensionless OD units for OD_600_).

The cross‐validated RMSE (RMSE_CV_) ${\mathrm{RMSE}}_{\mathrm{cv}}=\mathrm{mean}\;{\mathrm{RMSE}}_{\mathrm{folds}}$ was computed as the mean RMSE across all folds, and the standard deviation (SD_CV_) quantified batch‐to‐batch variability in model performance. The coefficient of determination (*R* ^2^_CV_) was similarly computed from out‐of‐fold predictions pooled across all batches. Exponential models were fitted using log‐transformation of the response variable to stabilize variance, with predictions back‐transformed to the original scale. Across the six selected models, heteroscedasticity was observed (variance ratios 4.2–566x between upper and lower tertiles of fitted values (Supporting Information S1: V‐Table S5 and Figure S4a–c), which is physically expected in optical density measurements at high cell concentrations due to multiple light scattering phenomena (Myers et al. 2013). The variance structure does not bias prediction accuracy: LOBO‐CV provides distribution‐free estimates of generalization error without requiring homoscedastic residuals (Hastie et al. 2009). Confidence intervals for RMSE_CV_ were computed from the empirical standard error across cross‐validation folds, following the framework for cross‐validation inference described by Nadeau and Bengio (2003).

#### 2.6.2 Model Selection and Evaluation

Final model selection employed a two‐stage procedure combining quantitative performance ranking with parsimony considerations (Hastie et al. 2009). First, models were ranked by RMSE_CV_. Second, a practical equivalence criterion was applied: models within 5% of the minimum RMSECV were considered practically equivalent and the simplest model (lowest EDF) was selected among equivalents. This threshold was selected based on typical gravimetric DCW measurement uncertainty (CV ~3%–5%; Kiviharju et al. 2007), reasoning that model differences smaller than analytical precision are not practically meaningful for bioprocess applications. The calculated 95% confidence intervals (Table 2) provide statistical support for the practical equivalence criterion. For models falling within this threshold, confidence intervals exhibit substantial overlap, confirming that observed performance differences are not statistically distinguishable from measurement variability. For example, in the *P. pastoris* DCW case, the selected models ns_4 (${\text{RMSE}}_{\text{CV}}=5.96,\;{\text{CI}}^{95}=\left[1.81,\;10.11\right]$) and bs_1 (${\text{RMSE}}_{\text{CV}}=6.31,\;{\text{CI}}^{95}=\left[1.57,\;11.05\right]$) show completely overlapping intervals despite their 5.9% difference in point estimates.

**Table 2:** Model Selection Performance. Predictive performance and selection of best regression models correlating online OD_860_ with offline biomass indicators DCW and OD_600_ across three industrially relevant yeast strains. The sensor type indicates whether the model is optimal for software‐based systems (software), calculator‐based systems (calculator), or both. RMSE_CV_ and *R* ^2^_CV_ represent leave‐one‐batch‐out cross‐validated metrics.

Yeast	Feature	Sensor Type	Model	RMSE_CV_	CI_95_ lower	CI_95_ upper	SD_CV_	*R* ^2^_CV_	EDF	BP (AU)
*Pichia pastoris*	DCW	Software	ns_4	5.96	1.81	10.11	2.99	0.914	5	N/A
		Calculator	bs_1	6.31	1.57	11.05	3.41	0.905	3	1.92
	OD_600_	Software	ns_3	29.03	11.46	46.60	12.66	0.895	4	N/A
		Calculator	bs_1	33.48	11.54	55.43	15.81	0.858	3	2.16
*Yarrowia lipolytica*	DCW	Both	Freundlich	13.06	4.91	21.20	8.15	0.853	2	N/A
	OD_600_	Both	Exponential	92.62	9.59	175.64	83.12	0.685	2	N/A
*Kluyveromyces marxianus*	DCW	Software	ns_3	5.72	3.07	8.37	2.65	0.855	4	N/A
		Calculator	bs_1	6.07	4.13	8.02	1.94	0.854	3	1.86
	OD_600_	Both	Exponential	52.73	6.11	99.34	46.66	0.489	2	N/A

*Note:* RMSE_CV: Root Mean Squared Error from nested leave‐one‐batch‐out cross‐validation. CI_95_: 95% confidence intervals calculated using the Nadeau and Bengio (2003) corrected variance estimator. SD_CV: Standard deviation across cross‐validation folds. *R* ^2^_CV: Coefficient of determination from cross‐validation. EDF: Effective degrees of freedom. Breakpoint: Optical density threshold (AU) separating quasi‐linear from nonlinear scattering regimes, calculated from final model fitted to complete dataset. Values represent the inflection point for piecewise models (bs_1‐4, ns_2_bp) that explicitly incorporate this breakpoint in their structure. For exponential and Freundlich models, breakpoint detection is not applicable (N/A). For continuous models (Freundlich, exponential), the BP does not enter the equation and is therefore reported as N/A in this table; the corresponding fold‐mean estimates from nested LOBO‐CV are reported in Supporting Information S1: Table S1 and used for mechanistic discussion in the text. Units: RMSE_CV, CI_95_, and SD_CV in g L^‐1^ for DCW, dimensionless OD units for OD_600_; Breakpoint in OD_860_ absorbance units (AU).

Sensitivity analysis across tolerance thresholds from 2.5% to 10% (Supporting Information S1: II) demonstrated robust model selection, with three of six cases showing complete stability across all thresholds (*P. pastoris* OD_600_, *Y. lipolytica* OD_600_ and *K. marxianus* OD_600_). The remaining three cases exhibited predictable transitions toward more parsimonious models at higher thresholds, confirming that the 5% tolerance represents a balanced choice.

Following model selection, final predictive equations were generated by refitting the selected model to the complete dataset. These refit coefficients are reported in Table 2 for practical implementation. Performance metrics from the refit models (RMSE_refit_, *R* ^2^_refit_) are provided for reference but should not be used for model comparison due to optimistic bias inherent in training‐set evaluation. To quantify uncertainty in cross‐validated performance metrics, 95% confidence intervals for RMSE_CV_ were calculated as

$${\mathrm{CI}}_{95}={\mathrm{RMSE}}_{\mathrm{CV}}\;\pm t\;\left(\frac{\alpha }{2}\;n-1\right)\times \mathrm{SE}\mathrm{\_corrected}.$$

where $\text{SE\_corrected}=\frac{{\text{SD}}_{\mathrm{CV}}}{\surd n}\times \surd \left(1+\frac{n_{\text{test}}}{n_{\text{train}}}\right)\;$is the standard error across the *k* = 5–7 cross‐validation folds, and t_0_._975_ denotes the upper 0.025 quantile of Student's t distribution. This empirical SE estimator follows the principles of cross‐validation inference described by Nadeau and Bengio (2003), without imposing parametric assumptions on the error distribution. In this notation, *n* = k denotes the number of cross‐validation folds (k = 5 for *P. pastoris*; *k* = 7 for *Y. lipolytica* and *K. marxianus*), with n_test = 1 batch held out per fold and n_train = k − 1, consistent with the LOBO design.

## 3 Results and Discussion

Linear regression (poly_1) achieved RMSE_CV_ of 9.8–20.3 g L^−1^ for DCW prediction across strains (Supporting Information S1: III), representing the baseline against which nonlinear models are evaluated. The selected nonlinear models reduced RMSE_CV_ by 36%–45% relative to this baseline, confirming the need to account for multiple‐scattering effects at high cell densities. Model uncertainty is quantified by the standard deviation across cross‐validation folds (SD_CV_, Table 2). For DCW prediction, SD_CV_ ranged from 1.9 to 8.2 g L^−1^, reflecting typical batch‐to‐batch variability and providing realistic expectations for prediction stability in industrial fermentation runs.

### 3.1 Data Characterization

**Figure 2:**
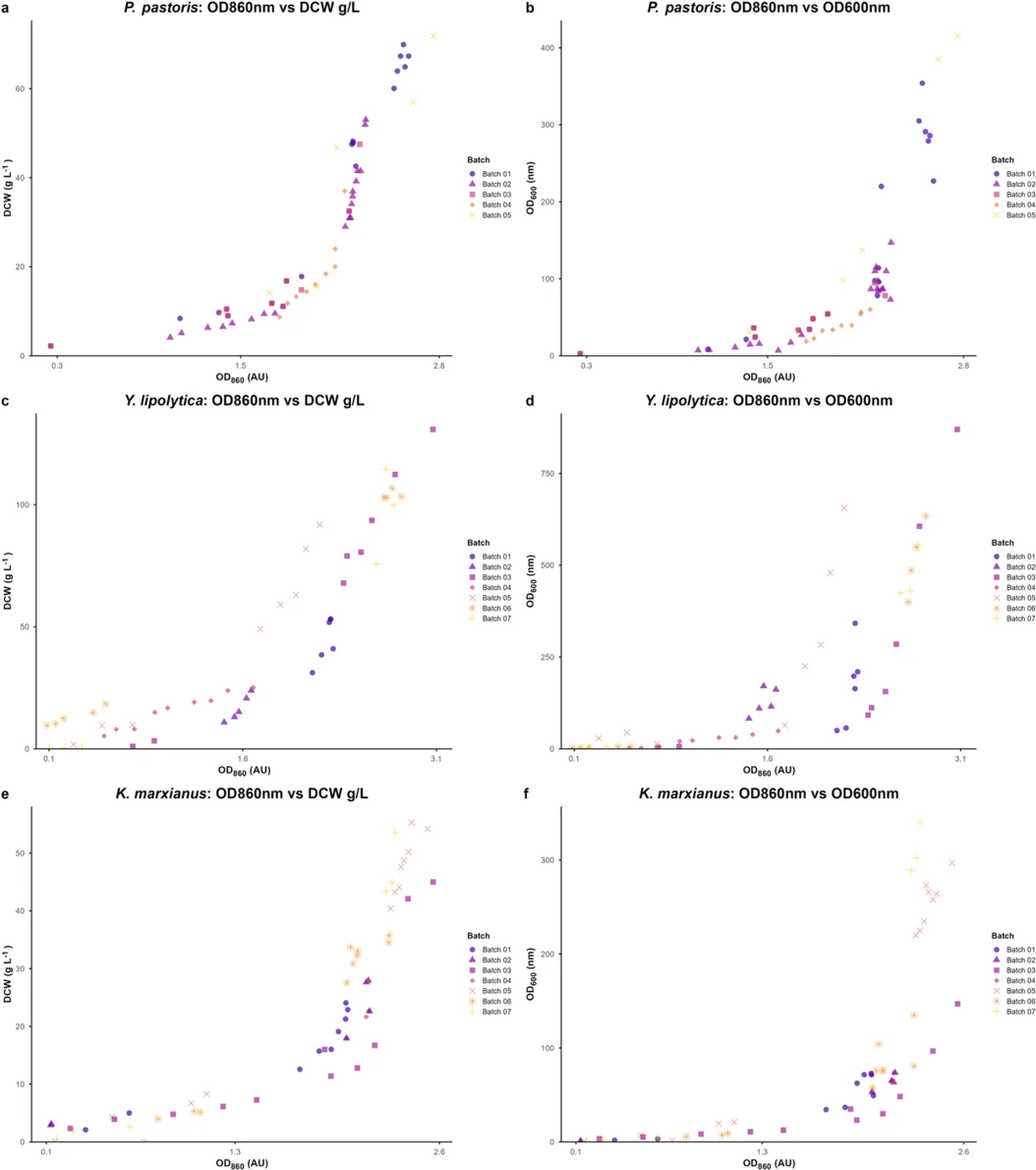
Relationships between online OD_860_ and offline DCW (left) and OD_600_ (right). Panels: (a and b) *Pichia pastoris*, (c and d) *Yarrowia lipolytica*, (e and f) *Kluyveromyces marxianus.* Offline indicators exhibited clear strain‐dependent differences: moderate biomass in *P. pastoris*, the highest accumulation in *Y. lipolytica*, and lower but faster growth rate in *K. marxianus*.

Initial data characterization confirmed the inherent difficulties of traditional biomass measurement methods and the need for soft sensor‐based estimation. Strain‐specific scatterplots (Figure 2) illustrate the relationships between OD_860_ and the offline indicators OD_600_ and DCW, highlighting two critical issues common to all three yeasts: offline variability and signal nonlinearity.

High‐density cultures often exceeded the spectrophotometer's linear detection range, requiring multiple dilutions that introduced significant measurement variability (Ji et al. 2024; Myers et al. 2013). Optical density measurements are susceptible to interference from metabolic pigments, gas bubbles, and antifoam agents (Biechele et al. 2015; Ulber et al. 2003). Additionally, optical density measurements are limited to recognizing viable and dead cells at the stationary phase (Casablancas et al. 2013). The continuous OD_860_signal, while robust, revealed fundamental deviations from linearity at high biomass densities (Figure 2). At high cell concentrations, the relationship between OD and cell mass is intrinsically nonlinear due to multiple light scattering inherent to dense suspensions (Kitchener et al. 2017; Luong et al. 2011). These effects arise from changes in cell morphology and refractive index (Myers et al. 2013), requiring robust, non‐linear mathematical models to map OD_860_ to reliable DCW and OD_600_ standards (Hastie et al. 2009; Kadlec et al. 2009).

### 3.2 Model Evaluation and Strain‐Specific Performance

**Figure 3:**
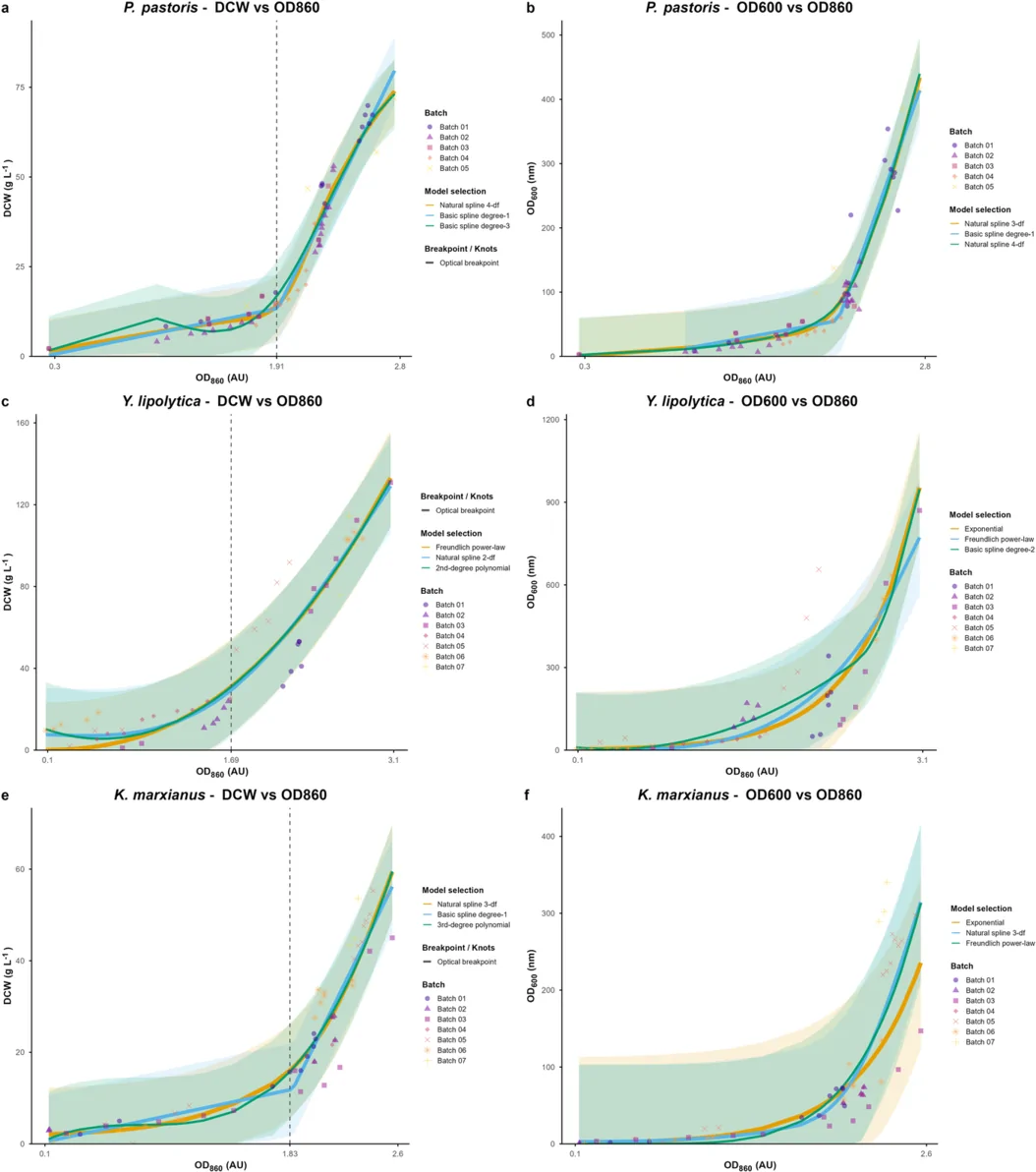
Top‐ranked regression models correlating the online OD_860_ signal (AU) with offline biomass indicators (DCW g L^−1^; OD_600_) for the three yeast strains: Panels: (a) *Pichia pastoris* OD_860_ vs. DCW; (b) *P. pastoris* OD_860_ vs. OD_600_; (c) *Y. lipolytica* OD_860_ vs. DCW; (d) *Yarrowia lipolytica* OD_860_ vs. OD_600_; (e) *Kluyveromyces marxianus* OD_860_ vs. DCW; (f) *K. marxianus* OD_860_ vs. OD_600_. Each panel shows three regression curves: the top two software‐ranked models and the calculator alternative selected for closed‐form predictions; the calculator curve is highlighted. Shaded bands represent the 95% confidence intervals derived from out‐of‐fold predictions. Dashed vertical lines in the DCW panels (a, c, e) mark the species‐specific optical breakpoints—the OD_860_ value at which the inline signal departs from a linear relationship with biomass concentration owing to the onset of multiple scattering (*P. pastoris* 1.91 AU; *Y. lipolytica* 1.69 AU; *K. marxianus* 1.83 AU; mean fold‐specific values from nested LOBO‐CV, Table S1; mechanistic interpretation in Section 3.4, 4). Breakpoints are not displayed on the OD_600_ panels (b, d, f), where both axes are optical observables and a single threshold does not correspond to a discrete physical transition.

Fourteen regression models were evaluated across the three yeast strains for both DCW and OD_600_ prediction using nested LOBO‐CV (Supporting Information S1: IV). Model selection was performed using the parsimony criterion with a 5% practical equivalence threshold. Table 2 summarizes the performance of selected models, including both software‐optimized and calculator‐compatible alternatives, and Figure 3 presents the best‐fitting regression curves overlaid on experimental data, with dashed vertical lines indicating strain‐specific optical breakpoints.

#### 3.2.1 Pichia pastoris

The recombinant *P. pastoris* with the pGAP promoter achieved moderate biomass levels, with maximum OD_600_ and DCW values of 415 and 71.8 g L^−1^, respectively, across five independent fed‐batch fermentations (63 observations total).

For DCW prediction, the natural spline with four degrees of freedom (ns_4) was selected as the software model, achieving RMSE_CV_ = 5.96 g L^−1^ (SD_CV_ = 2.99) and *R* ^2^_CV_ = 0.914. The basic spline degree 1 (bs_1) was selected as the calculator alternative (RMSE_CV_ = 6.31 g L^−1^), incurring a modest 5.8% accuracy penalty at the cost of a simple piecewise‐linear equation. The optical breakpoint of the bs_1 calculator equation was identified at 1.92 AU (Table 2), consistent with the transition to multiple light scattering at high cell densities.

For OD_600_ prediction, the natural spline with three degrees of freedom (ns_3) provided the optimal software model (RMSE_CV_ = 29.03, SD_CV_ = 12.66, *R* ^2^_CV_ = 0.895). The calculator alternative (bs_1, RMSE_CV_ = 33.48) incurred a 15.3% accuracy penalty, reflecting the greater nonlinearity in the OD_600_ response that natural splines capture more effectively. The breakpoint for OD_600_ prediction (2.16 AU) was slightly higher than for DCW, potentially reflecting differences in how dilution‐corrected spectrophotometric measurements respond to optical saturation. The lower *R* ^2^_CV_ for OD_600_ compared to DCW reflects the amplified measurement uncertainty in dilution‐corrected spectrophotometric measurements, where small pipetting errors at high dilution factors (up to 1:1000 at peak biomass) propagate substantially.

#### 3.2.2 Yarrowia lipolytica

The oleaginous yeast *Y. lipolytica* reached the highest biomass densities among the evaluated strains, reaching OD_600_ values up to 870 and DCW concentrations of 130.8 g L^−1^ across seven independent batches (51 observations total).

For DCW prediction, the Freundlich power‐law model was selected for both software and calculator implementations (RMSE_CV_ = 13.1 g L^−1^, SD_CV_ = 8.2, *R* ^2^_CV_ = 0.853). The fitted equation (DCW = 8.61 × OD_860_^2·44^) exhibits a supralinear exponent (2.44 > 1), physiologically interpretable as lipid‐induced amplification of light scattering: under nitrogen limitation, *Y. lipolytica* accumulates large, highly refractive lipid bodies that increase scattering per unit dry weight (Papanikolaou et al. 2001). The Freundlich model captures both the mathematical and physiological aspects of this behavior with only two parameters (EDF = 2), satisfying the parsimony criterion.

The optical breakpoints for *Y. lipolytica* were identified at 1.69 AU (DCW, CV = 19.8%) and 2.36 AU (OD_600_, CV = 17.9%), exhibiting higher variability than non‐oleaginous strains. The lower DCW breakpoint compared to *P. pastoris* (1.92 AU) and *K. marxianus* (1.86 AU) is consistent with lipid accumulation amplifying optical density per unit dry weight, causing the nonlinear scattering regime to commence at lower apparent cell densities. The differences between fold‐averaged breakpoints calculated during cross‐validation (Supporting Information S1: IB and Table S1) and final model breakpoints fitted to the complete data set are most pronounced for *Y. lipolytica* (0.14–0.16 AU), reflecting the high biological variability of this oleaginous, dimorphic yeast (Table 2). For *Y. lipolytica*, the value reported throughout the present study (1.69 AU) corresponds to the fold‐mean from nested LOBO‐CV (Supporting Information S1: Table S1), which is statistically more robust than a single full‐dataset estimate given the high inter‐batch variability (CV = 19.8%); since the selected Freundlich model is continuous and does not employ the breakpoint in its equation, this distinction is informative rather than operational.

For OD_600_ prediction, the exponential model was selected (OD_600_ = 2.65 × e^1.92 × OD^_860_), achieving RMSE_CV_ = 92.6 and *R* ^2^_CV_ = 0.685. Substantial batch‐to‐batch variability was observed (SD_CV_ = 83.1), attributed to the dimorphic nature of *Y. lipolytica* (yeast‐to‐mycelium transition) and variable intracellular lipid accumulation, affecting light scattering properties (Timoumi et al. 2023). The lower *R* ^2^_CV_ for OD_600_ compared to DCW reflects the amplified measurement uncertainty in dilution‐corrected spectrophotometric measurements, where small pipetting errors at high dilution factors (up to 1:1000 at peak biomass) propagate substantially.

#### 3.2.3 Kluyveromyces marxianus

*K. marxianus* displayed stable optical behavior across seven independent batches (59 observations), reaching maximum OD_600_ and DCW values of 340 and 55.3 g L^−1^, respectively. This stability is likely due to its relatively uniform cell size and low aggregation tendency (McCarthy et al. 2002). For DCW estimation, the natural spline with three degrees of freedom (ns_3) was selected as the software model (RMSE_CV_ = 5.72 g L^−1^, SD_CV_ = 2.65, *R* ^2^_CV_ = 0.855). The basic spline degree 1 (bs_1) was selected for calculator use (RMSE_CV_ = 6.07 g L^−1^), with a minimal 6.2% accuracy penalty. The low SD_CV_ indicates consistent model performance across batches, supporting reliable deployment. The optical breakpoint of the bs_1 calculator model fitted to the complete dataset (1.86 AU; Table 2) was comparable to that observed for *P. pastoris* (1.92 AU), indicating similar cell‐size distributions; the corresponding fold‐mean estimate from nested LOBO‐CV (1.83 AU; Supporting Information S1: Table S1) is reported in the Figure 3 caption.

For OD_600_ prediction, the exponential model was selected for both implementations (RMSE_CV_ = 52.7, SD_CV_ = 46.7, *R* ^2^_CV_ = 0.489). The lower *R* ^2^_CV_ compared to DCW prediction (0.855) reflects the dilution‐correction uncertainty described for *P. pastoris*. Despite the modest variance explained, the exponential model provides the most parsimonious fit among candidates within the tolerance threshold. The optical breakpoint for OD_600_ prediction (2.02 AU, CV = 3.7%) showed consistency with DCW, reflecting *K. marxianus'* stable optical behavior.

### 3.3 Model Selection and Parsimony

Final model selection prioritized parsimony, selecting the simplest model that captures the underlying relationship without fitting noise (Hastie et al. 2009). The parsimony criterion operationalized this principle by selecting simpler models (lower EDFs) when performance was practically equivalent to those of more complex alternatives.

In three of six cases, the optimal software and calculator implementations coincided: Freundlich for *Y. lipolytica* DCW, and exponential for OD_600_ in both *Y. lipolytica* and *K. marxianus*, confirming that closed‐form models can achieve competitive accuracy without piecewise structure. In the remaining three cases (*P. pastoris* DCW and OD_600_; *K. marxianus* DCW), natural splines provided superior software performance, while piecewise‐linear calculator alternatives incurred accuracy penalties of 5.8%–15.3%.

Spline‐based models prove effective because their piecewise structure explicitly captures the optical breakpoint, the transition from single to multiple light scattering that fundamentally changes the OD_860_‐biomass relationship (Luong et al. 2011). The identified breakpoints (1.69–2.36 AU across strains) are physically consistent with Mie scattering theory (Mayerhöfer et al. 2020): at cell diameters of 2.84–4.59 µm and *λ* = 860 nm, the size parameter *x* = πd/λ ranges from 10.4 to 16.8 (Table 5), placing all species in the regime where scattering is nonlinear. The calculator‐based equations (Table 3) encode this transition explicitly, giving operators an interpretable threshold between the quasi‐linear and nonlinear operating regimes.

**Table 3:** Final selected models for predicting biomass (DCW and OD600) prediction as a function of OD860 across three yeast species. For piecewise linear models (bs_1), OD_860_ values below the breakpoint uses the left equation and OD_860_ above the breakpoint uses the right equation. For continuous models (Freundlich, exponential), the BP value shown in parentheses is informative (mean fold‐specific from LOBO‐CV) and does not enter the equation; the same equation applies across the full range.

Yeast Strain	Output Variable	Model	BP (OD_860,_ AU)	Operational range (AU)	Model (x ≤ BP) x = OD_860_, AU	Model (x ≥ BP) x = OD_860_, AU
*Pichia pastoris*	DCW	Basic Spline‐1	1.92	0.26–2.76	y=−1.71+7.90×x	y=−136.89+78.46×x
	OD_600_	Basic Spline‐1	2.16		y=−28.31+38.86×x	y=−1,237.59+598.42×x
*Yarrowia lipolytica*	DCW	Freundlich	N/A	0.08–3.07	$y=8.61\;\times \;x^{2.44}$	N/A
	OD_600_	Exponential	N/A		$y=2.65\;\times \;e^{\left(1.92\times x\right)}$	N/A
*Kluyveromyces marxianus*	DCW	Basic Spline‐1	1.86	0.13–2.56	y=−0.21+6.54×x	y=−104.61+62.82×x
	OD_600_	Exponential	N/A		$y=0.87\;\times \;e^{\left(2.19\;\times x\right)}$	N/A

*Note:* Models are expressed as a function of OD_860_ (AU). For Basic Spline‐1 models, the breakpoint (BP) divides the operational range into two linear regimes. For Freundlich and Exponential models, the BP shown in parentheses is the mean fold‐specific value from LOBO‐CV (informative; the same equation applies across the full operational range). Operational range corresponds to the OD_860_ range observed in the training data; predictions outside this range constitute extrapolation. N/A: model defined over the full range without breakpoint.

The quasi‐linear regime for each species, defined by the species‐specific breakpoints reported in Table 2, extended to 1.92 AU (*P. pastoris*), 1.86 AU (*K. marxianus*), and 1.69 AU (*Y. lipolytica*) for DCW models (Table 2 and Supporting Information S1: S1). Within these ranges, the OD_860_ response was approximately linear for all three strains. However, statistically significant curvature was detected below the breakpoint for *P. pastoris* (*p* = 0.020) and *K. marxianus* (*p* = 0.002), assessed by the quadratic term in a localized polynomial regression, indicating early‐onset multiple scattering below the classical Beer–Lambert limit. *Y. lipolytica* showed no significant curvature below its breakpoint (*p* = 0.32), but exhibited a steep linear phase followed by abrupt deviation, consistent with a rapid transition into the multiple‐scattering regime, likely linked to its oleaginous intracellular composition. For *K. marxianus*, this early curvature explains why the selected nonlinear models outperformed a local linear calibration even within the quasi‐linear range, reducing out‐of‐fold RMSE by 9% (2.21 vs*.* 2.43 g L^−1^; Supporting information S1: Table S1B), while differences were small for *P. pastoris* (5.6% RMSE reduction; 2.51 vs*.* 2.66 g L^−1^) and *Y. lipolytica* exhibited a slight increase in local RMSE (7.92 vs*.* 7.32 g L^−1^), consistent with its more abrupt linear‐to‐nonlinear transition where a piecewise model gains less in the quasi‐linear range than for *K. marxianus*.

Above the breakpoint, the sensor operates in the multiple‐scattering regime where the Beer–Lambert linear relationship no longer holds. In this range, the nonlinear models achieved out‐of‐fold RMSE of 7.3–25.9 g L^−1^, effectively extending the monitored biomass range from 16 to 25 g L^−1^ at the breakpoint to 55–131 g L^−1^, a 252–421% increase in the operational DCW envelope without hardware modification.

Neither the breakpoint location (1.92, 1.86, and 1.69 AU; Table 2 and Supporting Information S1) nor the pre‐breakpoint slope was conserved across species. To test cross‐species universality, data from all three species below the most conservative common breakpoint (OD_860_ < 1.69 AU, *N* = 63) were pooled. The three‐species universal calibration yielded *R* ^2^ = 0.34 with a marginally non‐significant slope interaction (ANCOVA, *p* = 0.075) but a highly significant joint species effect (*F* = 13.66, *p* < 0.001), indicating that the *K. marxianus* and *P. pastoris* slopes are statistically similar to each other, while *Y. lipolytica* differs strongly from both. The low explained variance and the substantially steeper slope of *Y. lipolytica* render the pooled three‐species model inadequate for analytical purposes. A pooled *P. pastoris* + *K. marxianus* calibration achieved *R* ^2^ = 0.77 with no significant differences in slopes (*p* = 0.39) or intercepts (*p* = 0.24); species‐specific breakpoints yielded comparable results (interaction *p* = 0.305), confirming that a pooled baseline model is operationally viable for non‐oleaginous strains, albeit with a quantified precision penalty relative to strain‐specific calibrations (Supporting Information S1: Figure S2). *Y. lipolytica* exhibited a roughly twofold steeper slope (9.93 vs. 4.57–5.67 g L^−1^ AU^−1^), consistent with the biophysical mechanisms discussed in the following paragraphs.

Strain‐specific breakpoints identified through segmented regression provide insight into optical behavior under high‐density conditions. Above these thresholds, multiple scattering by neighboring particles redirects light along curved paths within the suspension, increasing the apparent optical pathlength and producing the sublinear OD_860_–DCW relationships observed above the species‐specific breakpoints (1.86–1.92 AU for non‐oleaginous yeasts; 1.69 AU for *Y. lipolytica*; Table 2 and Supporting Information S1). *P. pastoris* and *K. marxianus* exhibited remarkably consistent breakpoints (CV < 4%; Supporting Information S1: Table S1 and IB), reflecting their uniform, spherical to ellipsoidal cell morphology. In contrast, *Y. lipolytica* displayed substantially higher breakpoint variability (CV = 17.9%–19.8%) attributed to the two dimorphic yeast‐to‐mycelium transitions and variable intracellular lipid accumulation that create highly refractive lipid bodies, dramatically altering light‐scattering properties (Papanikolaou et al. 2001; Timoumi et al. 2017).

Breakpoint detection for OD_600_ prediction exhibited approximately 20%–26% higher variability (CV) than for DCW prediction in non‐oleaginous strains (*K. marxianus*: 3.7% vs. 3.1%; *P. pastoris*: 2.7% vs. 2.2%; Supporting Information S1: Table S1), consistent with the cumulative uncertainty of dilution‐correction procedures. For *Y. lipolytica*, both DCW and OD_600_ breakpoints showed similarly high variability (CV ~ 17.9%–19.8%), suggesting that morphological heterogeneity dominates over measurement technique effects.

For the piecewise‐linear calculator models (bs_1), the equation given in Table 3 is continuous at the breakpoint by construction: a B‐spline of degree 1 with a single internal knot is continuous through its basis functions, with the two linear pieces sharing a common value at the knot. When the fitted coefficients are truncated to two decimal places for tabulated presentation, a small numerical artefact of approximately 0.29 g L^−1^ (≈2.2% of the predicted value at the breakpoint for *P. pastoris* DCW) appears as a residual jump; this rounding‐induced gap is well within the ±5% practical equivalence threshold and the analytical variability of gravimetric DCW (typically a few percent), and is therefore operationally negligible. Higher‐order splines (bs_2, bs_3, natural splines) used as software models add continuity of first and higher derivatives (C^1^, C^2^), providing smoother transitions across the breakpoint at the cost of requiring spline‐aware software for evaluation rather than direct hand calculation; this trade‐off motivates the dual reporting of a software‐ranked top model and a closed‐form calculator alternative throughout this work.

The lower average breakpoint for *Y. lipolytica* (DCW 1.69 AU vs. 1.86–1.92 AU for non‐oleaginous yeasts) supports the hypothesis that lipid accumulation amplifies optical density per unit dry weight, causing the nonlinear scattering regime to commence at lower apparent cell densities. For industrial applications, bioprocess operators should use the calculator equations of Table 3 over their stated operational ranges. For *P. pastoris* and *K. marxianus*, the BP reported in Table 2 is the knot of the piecewise (bs_1) equation; for *Y. lipolytica*, the Freundlich and exponential models are continuous and apply across the full operational range without requiring a breakpoint.

### 3.4 Predictive Performance, Optical‐Mechanistic Interpretation, and Implications for Soft Sensor Application

Species‐dependent scattering behavior, not model failure, explains why a universal OD_860_‐biomass calibration does not hold across the three yeasts. Optical density at 860 nm responds to particle size, shape, and refractive‐index contrast at the cell–medium interface; dry cell weight (DCW) reflects total gravimetric mass regardless of cellular composition. These two quantities are therefore coupled only through cell morphology and intracellular composition, both of which vary substantially across species.

**Figure 4:**
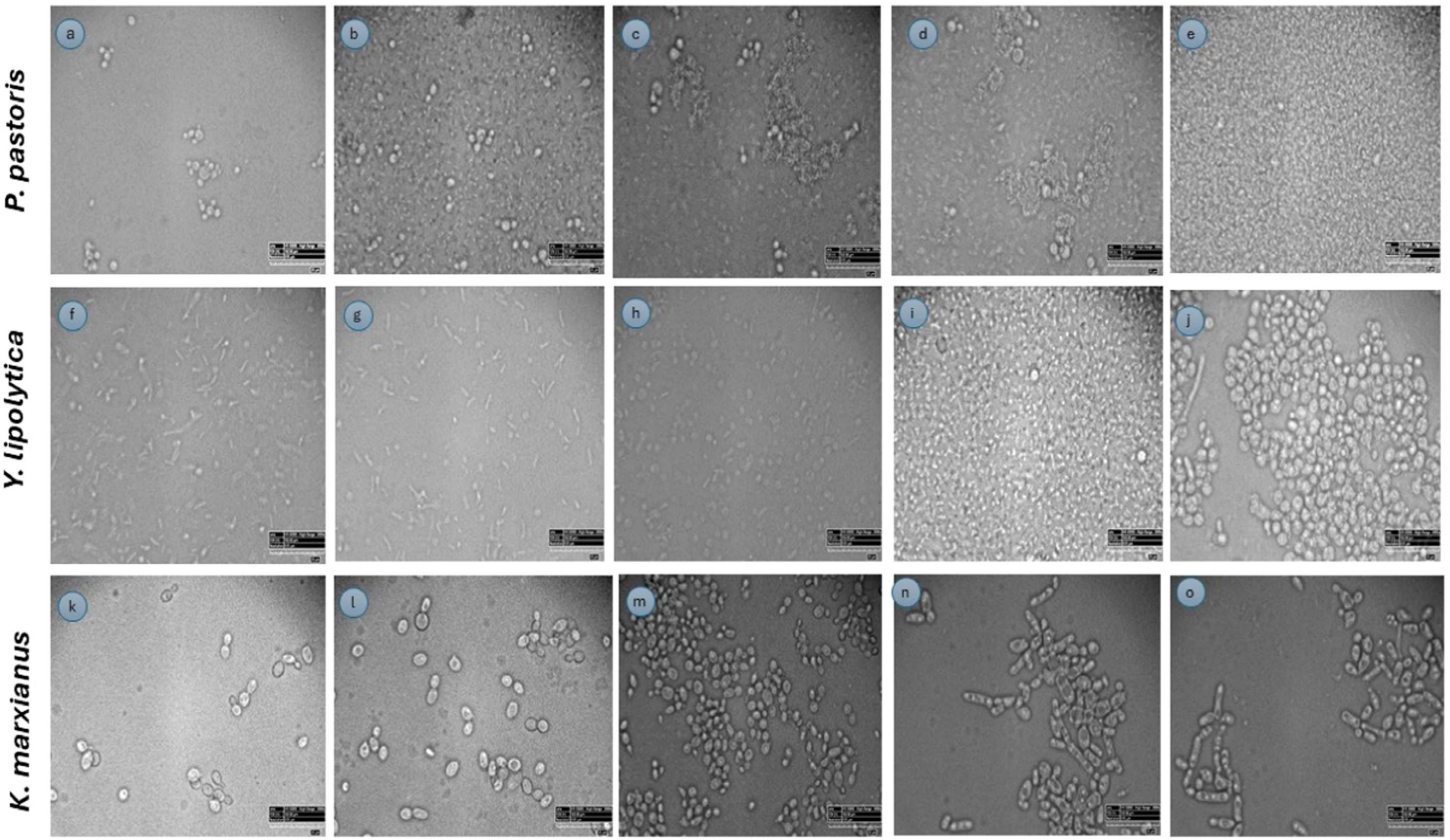
Representative bright‐field microscopy images showing the morphological progression of three yeast species (*Pichia pastoris*, *Yarrowia lipolytica*, and *Kluyveromyces marxianus*) at increasing OD_860_ values, used as a proxy for biomass concentration. (a–c) *P. pastoris* at 0.51, 1.33, and 1.50 AU; (d–f) *Y. lipolytica* at 0.51, 1.01, and 2.24 AU; (g–k) *K. marxianus* at 0.52, 0.98, 1.80, 2.44, and 2.70 AU. Scale bar: 20 µm. Images were obtained by brightfield microscopy; intracellular structures (lipid bodies in *Y. lipolytica*, budding scars in *K. marxianus*) are visible at higher magnification.

Brightfield microscopy at low cell densities (Table 4 and Figure 4) was performed in dedicated batches conducted for morphological analysis, separately from the 19 batches employed for soft sensor model training and validation. This separation was adopted to avoid invasive sampling of the modeling batches, which would have compromised the integrity of the paired OD_860_–DCW–OD_600_ time series.

**Table 4:** Cell morphometric measurements across OD_860_ sampling points for three yeast species. Cell size descriptors correspond to the Feret diameter (maximum caliper distance, µm) obtained from brightfield images via FIJI (Otsu thresholding, Analyze Particles, fitEllipse; scale 0.051 µm px^−1^). Median Feret diameter is reported with the cell‐by‐cell coefficient of variation (CV).

Band	Sample	Yeast	OD_860_ (AU)	*t* (h)	*n*	Median Feret (µm)	CV (%)	Q1 (µm)	Q3 (µm)	Range min–max (µm)	Morphology
Low	P1	*P. pastoris*	0.51	16	28	2.84	33.5	2.54	3.52	1.05–5.24	Ovoid
Mid‐low	P2	*P. pastoris*	1.33	22	26	3.64	26.0	2.84	3.99	1.60–4.89	Ovoid
Mid	P3	*P. pastoris*	1.50	20	21	3.19	24.8	2.89	4.09	1.50–4.89	Ovoid
Low	Y1	*Y. lipolytica*	0.51	5	10	3.34	25.4	2.82	4.46	2.59–4.79	Yeast‐form
Mid‐low	Y2	*Y. lipolytica*	1.01	7	10	3.39	18.7	2.82	3.82	2.29–4.09	Mixed
High	Y5	*Y. lipolytica*	2.24	22	9	4.59	18.5	3.69	5.29	3.09–5.34	Dimorphic
Low	K1	*K. marxianus*	0.52	5	14	4.14	20.6	3.79	4.84	3.59–6.53	Ovoid
Mid‐low	K2	*K. marxianus*	0.98	8	51	4.19	19.7	3.74	4.84	2.24–6.28	Ovoid
Mid‐high	K5	*K. marxianus*	1.80	10	10	4.59	38.9	4.26	5.84	3.74–10.62	Elongated
High	K3	*K. marxianus*	2.44	20	80	3.59	24.0	2.99	4.38	1.95–5.83	Ovoid‐elongated
High	K4	*K. marxianus*	2.70	26	83	4.19	19.7	3.44	4.64	2.84–6.13	Elongated

*Note: n* = number of individually resolvable cells. Microscopy samples were obtained from dedicated batches conducted ex profeso for morphological analysis (separate from the 19 batches used for soft sensor model training; Section 2.3). Sample numbering follows the original experimental sequence; OD_860_ values reflect the actual measurement at sampling time, with samples distributed across five density bands (Low, Mid‐low, Mid, Mid‐high, High).

The results revealed distinct morphological profiles at comparable OD_860_ values (~0.5 AU). *P. pastoris* cells were uniformly ovoid (median Feret 2.84 µm); *K. marxianus* cells were larger (median Feret 4.14 µm) with active budding; *Y. lipolytica* presented a yeast‐form morphology shifting to dimorphic at higher densities (median Feret 3.34–4.59 µm). With Feret diameters of 2.84–4.59 µm, all three species place the Mie size parameter (*x *= πd/λ) in the range 10.4–16.8 at 860 nm and 14.9–24.0 at 600 nm (Table 5), well within the regime where Beer–Lambert linearity fails at high cell density due to multiple scattering.

**Table 5:** Dimensionless size parameter (*x* = πd/λ) calculated from measured cell diameters across yeast species at *λ* = 860 and 600 nm. All values fall within the Mie scattering regime: *x* = 10.4–16.8 at *λ* = 860 nm and *x* = 14.9–24.0 at *λ* = 600 nm across the three species.

Species	Cell diameter (µm)	x (λ = 860 nm)	x (λ = 600 nm)	Optical Regime
*Pichia pastoris*	2.84–3.64	10.4–13.3	14.9–19.1	Mie regime
*Yarrowia lipolytica*	3.34–4.59	12.2–16.8	17.5–24.0	Mie regime
*Kluyveromyces marxianus*	3.59–4.59	13.1–16.8	18.8–24.0	Mie regime

*Note:* Cell diameter ranges correspond to *Median Feret* values reported in Table 4. The size parameter was calculated as *x * = πd/λ *where d is the median Feret diameter and λ is the wavelength of incident light. Values across both wavelengths confirm operation within the Mie scattering regime (x* = *10.4–16.8 at 860 nm; 14.9–24.0 at 600 nm)*.

In *P. pastoris*, the median Feret diameter remained stable between 2.84 and 3.64 µm across the measured density range, with cell‐by‐cell coefficient of variation (CV) decreasing from 33.5% (P1) to 24.8‐26.0% (P2‐P3). This morphological consistency produced a highly reproducible OD_860_–DCW relationship (breakpoint CV < 4% across batches) and the highest predictive accuracy of the three species (*R* ^2^CV = 0.914). *K. marxianus* showed larger cells (median Feret 4.14 µm at low density, increasing to 4.59 µm at intermediate densities; Lane et al. 2011). The cell‐by‐cell CV exhibited a non‐monotonic pattern: 20.6% at OD = 0.52 (K1), 19.7% at OD = 0.98 (K2), and 38.9% at OD = 1.80 (K5, dominated by elongated forms during active budding); above the breakpoint the CV decreased again to 24.0% (K3, OD = 2.44) and 19.7% (K4, OD = 2.70), consistent with cell elongation followed by population stabilization at higher densities. *Y. lipolytica* showed intermediate cell sizes that increased markedly above the breakpoint (median Feret 3.34 µm at OD_860_ = 0.51 AU, Y1; 3.39 µm at OD_860_ = 1.01 AU, Y2; 4.59 µm at OD_860_ = 2.24 AU, Y5), with cell‐by‐cell CV decreasing from 25.4% (Y1) to 18.5–18.7% (Y2, Y5) as the dimorphic yeast‐to‐mycelium transition progressed.

*Y. lipolytica* diverges from the two non‐oleaginous species through two concurrent biological mechanisms. First, dimorphic yeast‐to‐mycelium transitions are visible from the earliest sampling point (OD_860_ = 0.5 AU; Figure 4). Mycelial elements reduce the number of independent scattering particles per unit mass relative to an equivalent mass of discrete ovoid cells, decreasing optical attenuation per unit biomass (Timoumi et al. 2017). Intracellular lipid accumulation, a hallmark of oleaginous metabolism accounting for up to 20%–60% of DCW under nitrogen limitation (Beopoulos et al. 2009; Papanikolaou et al. 2001), alters the relationship between the optical signal and gravimetric mass through a thermodynamic mechanism: lipid droplets displace intracellular water, and because triglycerides do not volatilize at 80°C, lipid‐laden cells carry a higher dry‐mass‐to‐wet‐volume ratio than non‐oleaginous cells.

The OD_860_ probe responds to the geometry of hydrated cells, whereas DCW reflects the mass of desiccated pellets. These two factors, characterized by fewer scattering interfaces per unit mass and denser gravimetric mass per cell volume are consistent with the roughly twofold steeper DCW‐to‐OD_860_ slope + for this species (9.93 vs. 4.57–5.67 g L^−1^ AU^−1^) and its elevated batch‐to‐batch variability (SDCV = 8.15 g L^−1^). Although lipid bodies also carry a higher refractive index (n ≈ 1.48 vs. *n* ≈ 1.38 for cytoplasm; Beuthan et al. 1996), which increases localized scattering, this effect is counteracted at the macroscopic level by the geometric and mass‐density mechanisms described above. Intracellular lipid content was not measured directly in this study, but the magnitude of the cross‐species slope divergence substantially exceeds the analytical variability of the gravimetric DCW method (typically a few percent; (Kiviharju et al. 2007), and the same centrifugation and drying protocol was applied identically to all three species.

DCW prediction outperformed OD_600_ prediction across all species (*R* ^2^_CV_ = 0.85–0.91 vs. 0.49–0.90 and Table 2). Two independent factors drive this gap. First, OD_600_ measurements require serial dilution at high cell densities (up to 1:1000) to remain within the linear spectrophotometric range (< 0.9 AU). Small pipetting errors at these dilution factors propagate into large deviations in the corrected value. At OD_860_ ≥ 1.0 AU, the coefficient of variation of OD_600_ typically exceeded that of DCW by 1.5–2.6 across non‐oleaginous strains and at most density bins for *Y. lipolytica*; a single density window for *Y. lipolytica* (1.5–2.0 AU; ratio 0.81) showed the inverse pattern within this operational range, attributable to localized variability in the gravimetric reference at the onset of dimorphic transition. *K. marxianus* exhibited an additional inverse bin below the operational threshold (0.5–1.0 AU; ratio 0.93), where the gravimetric reference dominates the variance budget at low cell densities. DCW, determined gravimetrically, does not carry this concentration‐dependent amplification. Second, predicting OD_600_ from OD_860_ involves a statistical mapping between two distinct optical observables measured by different instruments, each with its own scattering response. At λ = 600 nm, the Mie size parameter for the measured cell diameters rises to x = 14.9–24.0 (Table 5), compared with x = 10.4–16.8 at 860 nm. Scattering efficiency is nonlinear and wavelength‐dependent throughout this range, introducing additional variance not present when predicting a wavelength‐independent gravimetric quantity.

Batch‐to‐batch variability, quantified as SD_CV_ (1.9–3.4 g L^−1^ for non‐oleaginous yeasts; 8.15 g L^−1^ for *Y. lipolytica*), provides a measure of prediction stability that is routinely omitted from soft sensor evaluations (Kadlec et al. 2009). For non‐oleaginous yeasts, the RMSE_CV_ values of 5.7–6.3 g L^−1^ are comparable to those reported by (Brunner et al. 2020), who combined off‐gas CO_2_ data with a phase‐detection algorithm for *P. pastoris* (pAOX1) and obtained RMSE = 5.05 g L^−1^ during the fed‐batch phase. The present models achieve similar fed‐batch accuracy using only OD_860_ as input, with 2–5 parameters rather than a hybrid stoichiometric architecture. For practical interpretation of real‐time sensor outputs, Table 6 provides representative DCW and OD_600_ values at OD_860_ = 1.0 and 2.0 AU. DCW prediction should be treated as the primary control variable. OD_600_ conversions serve as approximate reference values for operators accustomed to spectrophotometric units in process documentation.

**Table 6:** Calculated soft sensor prediction at Specific Online OD_860_ Values (DCW and OD600). Values represent predictions from calculator‐based models, providing a direct operational reference for correlating real‐time OD_860_ readings with conventional offline biomass indicators.

Yeast	OD_860_ (AU)	DCW (g L^−1^)	OD_600_
*Pichia pastoris*	1.0	6.19	10.55
	2.0	20.03	49.42
*Yarrowia lipolytica*	1.0	8.61	17.99
	2.0	46.68	122.15
*Kluyveromyces marxianus*	1.0	6.34	7.74
	2.0	21.03	69.14

The developed models enable non‐invasive, real‐time biomass estimation suitable for closed‐loop process control. The dual‐model strategy, combining a software‐optimized model for automated systems with simplified calculator‐based alternatives, extends deployment across control environments. This approach aligns with Process Analytical Technology (PAT) principles by providing real‐time, data‐driven biomass estimation in high‐density fermentation (Kadlec et al. 2009).

### 3.5 Limitations and Transferability of the OD_860_ Models

Among the model outputs, DCW prediction represents the most reliable basis for process monitoring and control, achieving *R* ^2^_CV_ = 0.85‐0.91 with SD_CV_ = 1.9–8.2 g L^−1^. Gravimetric DCW remains the standard quantitative metric for biomass concentration in high‐cell‐density fermentations and is directly relevant for mass balances, productivity calculations, and downstream processing decisions. It is important to note that gravimetric dry cell weight (DCW), while widely accepted as a reference method for biomass quantification, is not free from analytical variability. Reported uncertainties in DCW determination for yeast fermentations typically range within a few percent, depending on washing, drying protocol, and sample handling (Kiviharju et al. 2007). Minor deviations may arise from pellet handling, washing efficiency, residual bound water, or extracellular material.

Therefore, part of the apparent deviation between OD_860_ and DCW at high biomass concentrations may reflect intrinsic variability in the gravimetric reference itself rather than solely model limitations. Nevertheless, DCW remains the most direct and broadly accepted biomass metric in high‐cell‐density yeast fermentations, and the observed optical trends exceeded the magnitude expected from analytical DCW error alone. In contrast, OD_600_ predictions are inherently less accurate due to dilution‐driven noise amplification and wavelength‐dependent scattering effects (Section 3.4, 4). While included to facilitate comparison with standard laboratory practice, OD_600_ should be interpreted as an approximate conversion rather than a primary control variable.

Several limitations should be considered. First, the models were developed for a specific experimental configuration (3 L bioreactor, Hamilton Dencytee Arc probe at 860 nm with 5 mm optical path length, and defined media conditions). Scale‐up effects, including mixing heterogeneities and spatial concentration gradients, were not evaluated and may introduce systematic bias. Second, optical properties of yeast cells depend on growth conditions, and changes in morphology or intracellular composition may require recalibration. Third, the number of batches per strain (5–7) limits the precision of RMSE_CV_ estimates; industrial implementation should therefore include ongoing validation using new fermentation data. Finally, turbidity‐based measurements do not distinguish viable from non‐viable biomass, which may affect performance in long fermentations; integration with complementary methods such as capacitance‐based sensors may be required (Kiviharju et al. 2008). For applications requiring quantitative accuracy in process control decisions (feeding rate adjustments, harvest timing, scale‐up criteria), direct DCW prediction from OD_860_ is recommended as the primary soft sensor output. OD_600_ prediction models serve a complementary role as approximate conversion tools, enabling operators to interpret online OD_860_ readings in the spectrophotometric units widely used in process documentation and historical batch records, rather than as precision control instruments.

Transferability depends on several factors. Changes in probe geometry (optical path length or detection angle) directly affect the OD_860_–biomass relationship and require recalibration. Reactor scale can introduce spatial heterogeneities, while aeration, agitation, and antifoam strategies may affect signal stability through bubble formation or baseline drift. Medium composition can also influence optical signals, particularly in the presence of pigmentation or particulate matter. Among these factors, cell morphology is the dominant determinant of model performance and must be reassessed when strain, promoter system, or cultivation conditions change. As a practical guideline, operators should perform verification of sensor baseline stability (< 3% variation before and after cultivation), validation using a limited number of representative batches (3–5), and full recalibration if prediction errors exceed the cross‐validated RMSE values reported in Table 2.

Alternative soft sensor approaches based on off‐gas analysis or elemental balances are widely used for biomass estimation (Brunner et al. 2020; Jenzsch et al. 2006; Sagmeister et al. 2013). These methods rely on respiratory signals such as CO_2_ evolution and O_2_ uptake and can achieve high predictive accuracy when metabolic models are well parameterized. However, they require dedicated gas‐analysis hardware, reliable mass‐balance closure, and reparameterization when operating conditions change. In addition, off‐gas measurements are subject to temporal delays due to gas–liquid transfer and headspace dynamics.

Spectroscopic approaches (NIR or photon density wave spectroscopy) provide richer signal content but require more complex instrumentation and calibration (Biechele et al. 2015; Cervera et al. 2009; Schiewe et al. 2023). In contrast, the OD_860_‐based approach presented here relies on a single *in situ* optical signal combined with low‐dimensional nonlinear regression (2–5 parameters). The out‐of‐fold prediction errors obtained for non‐oleaginous yeasts (RMSE_CV_ = 5.7–6.3 g L^−1^; nested leave‐one‐batch‐out cross‐validation) are comparable to those reported for hybrid off‐gas models in *P. pastoris* (Brunner et al. 2020), which achieved 3.57 g L^−1^ for the entire process (nRMSE = 5.52%) and 5.05 g L^−1^ during the fed‐batch phase.

The primary advantage of the proposed approach lies not in model complexity, but in its low hardware requirements, instantaneous signal availability for process monitoring, and high interpretability. In addition, the explicit quantification of batch‐to‐batch variability and applicability across species with distinct optical properties supports its use as a practical and transferable soft‐sensing strategy. These features position the method as complementary, rather than a replacement for, more complex multi‐input approaches.

## 4 Conclusions

Single‐wavelength OD_860_ signals, measured with a standard NIR probe, can be converted into accurate real‐time biomass estimates across physiologically distinct industrial yeast species when species‐specific nonlinear models are used. Among 14 candidate models evaluated using nested leave‐one‐batch‐out cross‐validation, low‐dimensional models (2–5 parameters) achieved robust predictive performance (*R* ^2^ = 0.85–0.91) for DCW across *P. pastoris, K. marxianus*, and *Y. lipolytica*. A dual‐model strategy, combining software‐optimized implementation with simplified piecewise formulations, enables deployment across both automated control systems and manual operational settings.

Biomass calibration is inherently species‐specific. Differences in cell morphology and intracellular composition, from the morphologically stable ovoid form of *P. pastoris* to the progressive elongation of *K. marxianus* and the dimorphic, lipid‐rich phenotype of *Y. lipolytica*, produce distinct scattering signatures that preclude a universal calibration. A pooled model is operationally viable for the two non‐oleaginous strains (*R* ^2^ = 0.77, no significant difference in slopes or intercepts) but fails when *Y. lipolytica* is included (three‐species pooled *R* ^2^ = 0.34), where lipid accumulation and dimorphic growth fundamentally decouple optical and gravimetric biomass signals.

DCW prediction provides the most reliable output for quantitative monitoring and process control. OD_600_ predictions carry higher uncertainty due to dilution‐driven noise amplification and wavelength‐dependent scattering and should be treated as approximate conversion values rather than control targets. Pairing a software‐optimized spline or power‐law function with a simplified piecewise‐linear equation extends deployment across automated control systems and calculator‐based environments while maintaining interpretability.

Scale‐up validation, assessment of probe placement effects in larger vessels, and inclusion of morphology‐related descriptors for structurally variable species such as *Y. lipolytica* are the most relevant directions for future work. Integration of OD_860_‐based soft sensors into closed‐loop control systems represents a practical next step toward robust, real‐time bioprocess monitoring using standard industrial hardware.

## Author Contributions

**Ana G. Del Hierro:** methodology, investigation, sampling, analysis, figures design, analysis and writing. **José Luis Checa‐Barrera:** data validation, statistical analysis, modeling, writing and editing. **Juan A. Moreno‐Cid:** methodology, investigation, conceptualization, supervision. **Eoin Casey:** supervision, reviewing, and editing.

## Ethics Statement

This study did not involve human or animal subjects.

## Conflicts of Interest

The authors declare no conflicts of interest.

## Use of Artificial Intelligence Tools

The authors acknowledge the use of GPT‐4o (OpenAI) for assistance with English language editing, grammar, and stylistic consistency. The AI tool was not used to generate scientific content, data analysis, results, or interpretations. All scientific decisions, analyses, and conclusions are entirely the responsibility of the authors.

## Supporting information

Supporting File

## Data Availability

The data sets and analysis pipeline used for model development and validation are provided as Supporting Information S1: I–VI. The full data set (OD_860_, OD_600_, and DCW values for all yeast strains) and the annotated R analysis pipeline yeastODsoft_v1.0.0.R used to reproduce all figures, tables and quantitative claims of this manuscript are publicly available in the Zenodo repository (DOI:10.5281/zenodo.17540316).
