# Supplementary material for: Real‐Time Biomass Estimation in High‐Density Yeast Fermentations Using Soft Sensor Modeling
Source: Biotechnol Bioeng. 2026 Jun 11;123(9):2389–405. doi: 10.1002/bit.70266 (PMC13521467; doi:10.1002/bit.70266)
Supplement: Supplementary file 1 [file BIT-123-2389-s001.docx]

# SUPPORTING INFORMATION S1

**Real-time biomass estimation in high-density yeast fermentations using soft-sensor modeling**

Ana G. Del Hierro^1,2,3^, José Luis Checa-Barrera^3^, Juan A. Moreno-Cid ^3,4^ & Eoin Casey^1,2*^

1. School of Chemical and Bioprocess Engineering, University College Dublin, Belfield, Dublin, Ireland.
2. BiOrbic, Research Ireland Centre for Bioeconomy, Dublin, Ireland.
3. Bioprocessing R&D Department, Bionet, Parque Tecnológico Fuente Álamo, Fuente Álamo, Spain.
4. R&D Department, Probelte SAU- Agronova Biotech, 30500 Molina de Segura, Spain

***Corresponding Author**: Eoin Casey e-mail: [eoin.casey@ucd.ie](mailto:eoin.casey@ucd.ie)

# IA: Raw Paired Online-Offline Datasets

Complete experimental dataset containing 173 paired measurements of online optical density at 860 nm (OD₈₆₀) and offline biomass indicators (dry cell weight and optical density at 600 nm) across 19 independent fed-batch fermentations of three industrially relevant yeast species (Excel file). Brightfield micrographs provided as separate PDF.

# IB: Breakpoint Variability Across Cross-Validation Folds

Systematic quantification of breakpoint detection consistency across cross-validation folds. BP_mean represents the average optical density threshold (AU) across all folds, BP_SD quantifies absolute variability, and BP_CV (coefficient of variation) enables comparison across scales. Low CV values (<5%) for *P. pastoris* and *K. marxianus* confirm robust detection of the optical transition point, while higher variability in Y. lipolytica reflects its dimorphic nature and lipid-dependent optical properties.

**Table S1**. Optical Breakpoint Stability Across Nested LOBO-CV Folds

| **Yeast** | **Feature** | **Bp_Mean** | **Bp_Sd** | **Bp_Cv** | **N_Batches** |
| --- | --- | --- | --- | --- | --- |
| *P. pastoris* | DCW | 1.91 | 0.04 | 2.2 | 5 |
| *P. pastoris* | OD_600_ | 2.17 | 0.06 | 2.7 | 5 |
| *Y. lipolytica* | DCW | 1.69 | 0.33 | 19.8 | 7 |
| *Y. lipolytica* | OD_600_ | 2.36 | 0.42 | 17.9 | 7 |
| *K. marxianus* | DCW | 1.83 | 0.06 | 3.1 | 7 |
| *K. marxianus* | OD_600_ | 2.02 | 0.07 | 3.7 | 7 |

**Table S1B. Prediction performance by sensor regime (DCW**). Out-of-fold predictions partitioned at species-specific breakpoints listed in the Breakpoint column (see footnote for estimator details). Poly₁: out-of-fold RMSE from LOBO-CV (linear fit on below-breakpoint training data only). Proposed model RMSE (g L⁻¹): out-of-fold RMSE of the selected software model re-fit on the below-breakpoint data (partitioned LOBO-CV). RMSE above BP: out-of-fold RMSE for predictions above the species-specific breakpoint. Extension (%): operational range extension above breakpoint relative to the linear regime.

| **Species** | **Breakpoint (AU)** | **Poly₁ RMSE below BP (g L⁻¹)** | **Proposed model RMSE below BP (g L⁻¹)** | **Proposed model RMSE above BP (g L⁻¹)** | **Extension (%)** |
| --- | --- | --- | --- | --- | --- |
| *P. pastoris* | 1.92 | 2.66 | 2.51 | 7.34 | 304 |
| *K. marxianus* | 1.86 | 2.43 | 2.21 | 8.51 | 252 |
| *Y. lipolytica* | 1.69 | 7.32 | 7.92 | 25.89 | 421 |
| ¹ Poly₁: out-of-fold RMSE from LOBO-CV (linear fit on below-breakpoint training data only). Proposed model RMSE: out-of-fold RMSE from partitioned LOBO-CV — the selected software model (Table 2) is re-fit independently on the below- and above-breakpoint data, so this partitioned value differs from the full-range RMSE_CV reported in Table 2. Both metrics are computed out-of-fold, enabling direct comparison on unseen data.  Extension = (DCW_max − DCW_at_BP) / DCW_at_BP × 100. Breakpoints match those reported in Table 2 of the manuscript: for *P. pastoris* and *K. marxianus*, BP is the knot of the bs_1 calculator equation (full-dataset segmented regression); for *Y. lipolytica*, BP is the LOBO fold-mean (Table S1), reported in the manuscript because the full-dataset estimate is statistically less stable for this dimorphic, oleaginous yeast (CV = 19.8%). | | | | | |

**Figure S2. Quasi-linear regime comparison across species**. Data points represent individual observations below the most conservative common breakpoint (OD₈₆₀ < 1.69 AU; N = 63). Solid lines show species-specific linear regressions; dashed grey line shows the pooled *P. pastoris + K. marxianus* calibration (R² = 0.77, slopes p = 0.39, intercepts p = 0.24). The roughly twofold steeper slope of *Y. lipolytica* (9.93 vs 4.57–5.67 g L⁻¹ AU⁻¹) renders the three-species universal model inadequate (R² = 0.34). Shaded band: 95% confidence interval of the pooled regression.

# II: Tolerance Sensitivity Analysis

Model selection robustness analysis across different parsimony tolerance thresholds (2.5%, 5.0%, 7.5%, 10.0%). Demonstrates that the 5% tolerance threshold provides a balanced trade-off between predictive accuracy and model parsimony, with model selection remaining stable across most cases.

**Table S2. Model Selection at Different Tolerance Thresholds**

| **Case** | **Tolerance_Threshold %** | **Selected_Model** | **Rmse_Cv** | **R^2^_Cv** | **Edf** | **n_within_Tolerance** |
| --- | --- | --- | --- | --- | --- | --- |
| *P.pastoris_DCW* | 2.5 | ns_4 | 5.96 | 0.914 | 5 | 1 |
| *P. pastoris_DCW* | 5 | ns_4 | 5.96 | 0.914 | 5 | 1 |
| *P. pastoris_DCW* | 7.5 | bs_1 | 6.31 | 0.905 | 3 | 2 |
| *P. pastoris_DCW* | 10 | bs_1 | 6.31 | 0.905 | 3 | 3 |
| *P. pastoris_OD600* | 2.5 | ns_3 | 29.0 | 0.895 | 4 | 1 |
| *P. pastoris_OD600* | 5 | ns_3 | 29.0 | 0.895 | 4 | 1 |
| *P. pastoris_OD600* | 7.5 | ns_3 | 29.0 | 0.895 | 4 | 2 |
| *P. pastoris_OD600* | 10 | ns_3 | 29.0 | 0.895 | 4 | 2 |
| *Y. lipolytica_DCW* | 2.5 | ns_2 | 12.6 | 0.864 | 3 | 2 |
| *Y. lipolytica_DCW* | 5 | freundlich | 13.1 | 0.853 | 2 | 3 |
| *Y. lipolytica_DCW* | 7.5 | freundlich | 13.1 | 0.853 | 2 | 4 |
| *Y. lipolytica_DCW* | 10 | freundlich | 13.1 | 0.853 | 2 | 7 |
| *Y. lipolytica_OD600* | 2.5 | exponential | 92.6 | 0.685 | 2 | 1 |
| *Y. lipolytica_OD600* | 5 | exponential | 92.6 | 0.685 | 2 | 1 |
| *Y. lipolytica_OD600* | 7.5 | exponential | 92.6 | 0.685 | 2 | 1 |
| *Y. lipolytica_OD600* | 10 | exponential | 92.6 | 0.685 | 2 | 3 |
| *K. marxianus_DCW* | 2.5 | ns_3 | 5.72 | 0.855 | 4 | 4 |
| *K. marxianus_DCW* | 5 | ns_3 | 5.72 | 0.855 | 4 | 6 |
| *K. marxianus_DCW* | 7.5 | freundlich | 6.14 | 0.844 | 2 | 9 |
| *K. marxianus_DCW* | 10 | freundlich | 6.14 | 0.844 | 2 | 11 |
| *K. marxianus_OD600* | 2.5 | exponential | 52.7 | 0.489 | 2 | 1 |
| *K. marxianus_OD600* | 5 | exponential | 52.7 | 0.489 | 2 | 1 |
| *K. marxianus_OD600* | 7.5 | exponential | 52.7 | 0.489 | 2 | 1 |
| *K. marxianus_OD600* | 10 | exponential | 52.7 | 0.489 | 2 | 2 |

**Key Observations:**

Three cases show complete stability: *P. pastoris* OD₆₀₀ (ns_3), *Y. lipolytica* OD₆₀₀ (exponential), *K. marxianus* OD₆₀₀ (exponential). Remaining cases exhibit predictable transitions toward more parsimonious models at higher thresholds. The 5% tolerance represents typical gravimetric measurement uncertainty (CV ~3–5% for DCW assays)

# III: Baseline Linear Regression Performance

Simple linear regression (polynomial degree 1) performance metrics demonstrating the necessity of nonlinear modeling for accurate biomass estimation at high cell densities. The selected nonlinear models reduced RMSE_CV by 36–45% relative to this baseline.

**Table S3. Linear Baseline Model Performance**

| **Yeast** | **Feature** | **Rmse_Cv** | **Sd_Cv** | **R2_Cv** |
| --- | --- | --- | --- | --- |
| *P. pastoris* | DCW | 10.8 | 3.51 | 0.683 |
| *P. pastoris* | OD_600_ | 75.6 | 37.3 | 0.393 |
| *Y. lipolytica* | DCW | 20.3 | 6.49 | 0.713 |
| *Y. lipolytica* | OD_600_ | 143 | 57.8 | 0.511 |
| *K. marxianus* | DCW | 9.79 | 3.54 | 0.569 |
| *K. marxianus* | OD_600_ | 88.8 | 50.2 | 0.107 |

**Performance Comparison:**

*P. pastoris* DCW: Linear RMSE = 10.8 g L^-1^ vs. Nonlinear RMSE = 6.0 g L^-1^ (44.9% reduction)

*Y. lipolytica* DCW: Linear RMSE = 20.3 g L^-1^ vs. Nonlinear RMSE = 13.1 g L^-1^ (35.8% reduction)

*K. marxianus* DCW: Linear RMSE = 9.8 g L^-1^ vs. Nonlinear RMSE = 5.7 g L^-1^ (41.6% reduction)

Lower R²_CV values for OD₆₀₀ prediction reflect cumulative uncertainty from dilution-correction procedures

**Residual Plots**

Visual diagnostics for each yeast species showing residuals vs. fitted values and scale-location plots. The following three figures are provided as separate PDF files. **Each figure contains four panels:** Top-left: Residuals vs. fitted values for DCW prediction. Top-right: Scale-location plot for DCW (√|residuals| vs. fitted). Bottom-left: Residuals vs. fitted values for OD₆₀₀ prediction. Bottom-right: Scale-location plot for OD₆₀₀ (√|residuals| vs. fitted)

**Figure S4a. *P. pastoris* residual diagnostics.** Visual diagnostics showing residuals vs. fitted values and scale-location plots for the soft-sensor models. The four panels are: (top-left) residuals vs. fitted values for DCW prediction; (top-right) scale-location plot for DCW (√|residuals| vs. fitted); (bottom-left) residuals vs. fitted values for OD₆₀₀ prediction; (bottom-right) scale-location plot for OD₆₀₀ (√|residuals| vs. fitted).

***Figure S4b. Y. lipolytica residual diagnostics.*** *Visual diagnostics showing residuals vs. fitted values and scale-location plots for the soft-sensor models. The four panels are: (top-left) residuals vs. fitted values for DCW prediction; (top-right) scale-location plot for DCW (√|residuals| vs. fitted); (bottom-left) residuals vs. fitted values for OD₆₀₀ prediction; (bottom-right) scale-location plot for OD₆₀₀ (√|residuals| vs. fitted).*

**Figure S4c. *K. marxianus* residual diagnostics.** Visual diagnostics showing residuals vs. fitted values and scale-location plots for the soft-sensor models. The four panels are: (top-left) residuals vs. fitted values for DCW prediction; (top-right) scale-location plot for DCW (√|residuals| vs. fitted); (bottom-left) residuals vs. fitted values for OD₆₀₀ prediction; (bottom-right) scale-location plot for OD₆₀₀ (√|residuals| vs. fitted).

**IV: Complete Model Performance Summary**

Comprehensive cross-validated performance metrics for all 14 candidate regression models evaluated across six yeast-feature combinations using nested leave-one-batch-out cross-validation (LOBO-CV). This complete ranking demonstrates the systematic model selection process and quantifies performance trade-offs between accuracy and complexity.

**Table S4:** Cross-validated performance metrics for all 14-candidate regression

| **Yeast** | **Feature** | **Model** | **Rmse_Cv** | **Ci_Lower_95** | **Ci_Upper_95** | **Sd_Cv** | **R2_Cv** | **N_Converged** | **N_Folds** | **Case** | **Rank** | **Rmse_Penalty_Vs_Best_Pct** |
| --- | --- | --- | --- | --- | --- | --- | --- | --- | --- | --- | --- | --- |
| *P. pastoris* | DCW | poly_1 | 10.8 | 5.94 | 15.7 | 3.51 | 0.683 | 5 | 5 | *P. pastoris_DCW* | 14 | 81.50 |
| *P. pastoris* | DCW | poly_2 | 7.37 | 4.34 | 10.4 | 2.19 | 0.869 | 5 | 5 | *P. pastoris_DCW* | 10 | 23.76 |
| *P. pastoris* | DCW | poly_3 | 7.64 | 1.23 | 14.1 | 4.62 | 0.868 | 5 | 5 | *P. pastoris_DCW* | 12 | 28.27 |
| *P. pastoris* | DCW | poly_4 | 7.11 | 2.41 | 11.8 | 3.38 | 0.885 | 5 | 5 | *P. pastoris_DCW* | 7 | 19.27 |
| *P. pastoris* | DCW | exponential | 7.20 | 4.08 | 10.3 | 2.25 | 0.860 | 5 | 5 | *P. pastoris_DCW* | 8 | 20.81 |
| *P. pastoris* | DCW | freundlich | 7.59 | 1.75 | 13.4 | 4.21 | 0.870 | 5 | 5 | *P. pastoris_DCW* | 11 | 27.48 |
| *P. pastoris* | DCW | bs_1 | 6.31 | 1.57 | 11.0 | 3.41 | 0.905 | 5 | 5 | *P. pastoris_DCW* | 2 | 5.84 |
| *P. pastoris* | DCW | bs_2 | 7.90 | 1.66 | 14.1 | 4.49 | 0.860 | 5 | 5 | *P. pastoris_DCW* | 13 | 32.68 |
| *P. pastoris* | DCW | bs_3 | 6.46 | 2.69 | 10.2 | 2.71 | 0.905 | 5 | 5 | *P. pastoris_DCW* | 3 | 8.40 |
| *P. pastoris* | DCW | bs_4 | 6.60 | 0.00 | 13.8 | 5.15 | 0.899 | 5 | 5 | *P. pastoris_DCW* | 4 | 10.86 |
| *P. pastoris* | DCW | ns_2_bp | 6.89 | 3.74 | 10.0 | 2.26 | 0.891 | 5 | 5 | *P. pastoris_DCW* | 6 | 15.60 |
| *P. pastoris* | DCW | ns_2 | 6.72 | 3.49 | 9.94 | 2.32 | 0.896 | 5 | 5 | *P. pastoris_DCW* | 5 | 12.73 |
| *P. pastoris* | DCW | ns_3 | 7.35 | 2.05 | 12.7 | 3.82 | 0.880 | 5 | 5 | *P. pastoris_DCW* | 9 | 23.40 |
| *P. pastoris* | DCW | ns_4 | 5.96 | 1.81 | 10.1 | 2.99 | 0.914 | 5 | 5 | *P. pastoris_DCW* | 1 | 0.00 |
| *P. pastoris* | OD600 | poly_1 | 75.6 | 23.8 | 127 | 37.3 | 0.393 | 5 | 5 | *P. pastoris_OD600* | 14 | 160.54 |
| *P. pastoris* | OD600 | poly_2 | 60.1 | 37.4 | 82.8 | 16.4 | 0.661 | 5 | 5 | *P. pastoris_OD600* | 13 | 106.98 |
| *P. pastoris* | OD600 | poly_3 | 33.3 | 18.7 | 47.8 | 10.5 | 0.868 | 5 | 5 | *P. pastoris_OD600* | 3 | 14.65 |
| *P. pastoris* | OD600 | poly_4 | 33.4 | 14.0 | 52.7 | 13.9 | 0.866 | 5 | 5 | *P. pastoris_OD600* | 4 | 14.89 |
| *P. pastoris* | OD600 | exponential | 46.2 | 0.00 | 96.0 | 35.9 | 0.698 | 5 | 5 | *P. pastoris_OD600* | 10 | 59.00 |
| *P. pastoris* | OD600 | freundlich | 33.6 | 16.4 | 50.8 | 12.4 | 0.866 | 5 | 5 | *P. pastoris_OD600* | 6 | 15.71 |
| *P. pastoris* | OD600 | bs_1 | 33.5 | 11.5 | 55.4 | 15.8 | 0.858 | 5 | 5 | *P. pastoris_OD600* | 5 | 15.35 |
| *P. pastoris* | OD600 | bs_2 | 34.8 | 13.2 | 56.4 | 15.6 | 0.847 | 5 | 5 | *P. pastoris_OD600* | 7 | 19.79 |
| *P. pastoris* | OD600 | bs_3 | 37.5 | 16.5 | 58.5 | 15.1 | 0.832 | 5 | 5 | *P. pastoris_OD600* | 8 | 29.19 |
| *P. pastoris* | OD600 | bs_4 | 45.5 | 0.00 | 107 | 44.4 | 0.760 | 5 | 5 | *P. pastoris_OD600* | 9 | 56.63 |
| *P. pastoris* | OD600 | ns_2_bp | 54.5 | 32.5 | 76.6 | 15.9 | 0.722 | 5 | 5 | *P. pastoris_OD600* | 11 | 87.89 |
| *P. pastoris* | OD600 | ns_2 | 57.1 | 32.9 | 81.4 | 17.5 | 0.692 | 5 | 5 | *P. pastoris_OD600* | 12 | 96.82 |
| *P. pastoris* | OD600 | ns_3 | 29.0 | 11.5 | 46.6 | 12.7 | 0.895 | 5 | 5 | *P. pastoris_OD600* | 1 | 0.00 |
| *P. pastoris* | OD600 | ns_4 | 30.9 | 15.9 | 45.8 | 10.8 | 0.884 | 5 | 5 | *P. pastoris_OD600* | 2 | 6.27 |
| *Y. lipolytica* | DCW | poly_1 | 20.3 | 13.8 | 26.8 | 6.49 | 0.713 | 7 | 7 | *Y. lipolytica_DCW* | 14 | 61.77 |
| *Y. lipolytica* | DCW | poly_2 | 12.8 | 5.26 | 20.3 | 7.53 | 0.866 | 7 | 7 | *Y. lipolytica_DCW* | 2 | 1.70 |
| *Y. lipolytica* | DCW | poly_3 | 13.6 | 5.01 | 22.1 | 8.56 | 0.842 | 7 | 7 | *Y. lipolytica_DCW* | 5 | 7.95 |
| *Y. lipolytica* | DCW | poly_4 | 14.0 | 4.95 | 23.0 | 9.02 | 0.830 | 7 | 7 | *Y. lipolytica_DCW* | 8 | 11.13 |
| *Y. lipolytica* | DCW | exponential | 14.6 | 6.14 | 23.0 | 8.45 | 0.799 | 7 | 7 | *Y. lipolytica_DCW* | 10 | 16.00 |
| *Y. lipolytica* | DCW | freundlich | 13.1 | 4.91 | 21.2 | 8.15 | 0.853 | 7 | 7 | *Y. lipolytica_DCW* | 3 | 3.94 |
| *Y. lipolytica* | DCW | bs_1 | 16.8 | 6.51 | 27.0 | 10.3 | 0.760 | 7 | 7 | *Y. lipolytica_DCW* | 13 | 33.34 |
| *Y. lipolytica* | DCW | bs_2 | 13.6 | 5.13 | 22.1 | 8.47 | 0.844 | 7 | 7 | *Y. lipolytica_DCW* | 6 | 8.18 |
| *Y. lipolytica* | DCW | bs_3 | 14.1 | 4.57 | 23.7 | 9.58 | 0.820 | 7 | 7 | *Y. lipolytica_DCW* | 9 | 12.58 |
| *Y. lipolytica* | DCW | bs_4 | 16.5 | 7.25 | 25.7 | 9.25 | 0.772 | 7 | 7 | *Y. lipolytica_DCW* | 12 | 31.27 |
| *Y. lipolytica* | DCW | ns_2_bp | 13.2 | 5.24 | 21.2 | 8.01 | 0.853 | 7 | 7 | *Y. lipolytica_DCW* | 4 | 5.38 |
| *Y. lipolytica* | DCW | ns_2 | 12.6 | 4.55 | 20.6 | 8.02 | 0.864 | 7 | 7 | *Y. lipolytica_DCW* | 1 | 0.00 |
| *Y. lipolytica* | DCW | ns_3 | 13.7 | 4.46 | 22.9 | 9.24 | 0.832 | 7 | 7 | *Y. lipolytica_DCW* | 7 | 8.98 |
| *Y. lipolytica* | DCW | ns_4 | 15.0 | 5.20 | 24.9 | 9.85 | 0.808 | 7 | 7 | *Y. lipolytica_DCW* | 11 | 19.67 |
| *Y. lipolytica* | OD600 | poly_1 | 143 | 85.6 | 201 | 57.8 | 0.511 | 7 | 7 | *Y. lipolytica_OD600* | 14 | 54.84 |
| *Y. lipolytica* | OD600 | poly_2 | 107 | 29.6 | 185 | 77.8 | 0.660 | 7 | 7 | *Y. lipolytica_OD600* | 11 | 15.89 |
| *Y. lipolytica* | OD600 | poly_3 | 105 | 28.3 | 182 | 76.9 | 0.627 | 7 | 7 | *Y. lipolytica_OD600* | 6 | 13.53 |
| *Y. lipolytica* | OD600 | poly_4 | 105 | 18.7 | 192 | 86.6 | 0.649 | 7 | 7 | *Y. lipolytica_OD600* | 7 | 13.58 |
| *Y. lipolytica* | OD600 | exponential | 92.6 | 9.59 | 176 | 83.1 | 0.685 | 7 | 7 | *Y. lipolytica_OD600* | 1 | 0.00 |
| *Y. lipolytica* | OD600 | freundlich | 101 | 14.9 | 186 | 85.8 | 0.648 | 7 | 7 | *Y. lipolytica_OD600* | 2 | 8.62 |
| *Y. lipolytica* | OD600 | bs_1 | 102 | 14.8 | 189 | 87.3 | 0.603 | 7 | 7 | *Y. lipolytica_OD600* | 4 | 10.23 |
| *Y. lipolytica* | OD600 | bs_2 | 101 | 26.3 | 176 | 74.7 | 0.693 | 7 | 7 | *Y. lipolytica_OD600* | 3 | 8.97 |
| *Y. lipolytica* | OD600 | bs_3 | 106 | 31.5 | 180 | 74.4 | 0.652 | 7 | 7 | *Y. lipolytica_OD600* | 10 | 14.24 |
| *Y. lipolytica* | OD600 | bs_4 | 111 | 19.6 | 203 | 91.6 | 0.618 | 7 | 7 | *Y. lipolytica_OD600* | 12 | 19.94 |
| *Y. lipolytica* | OD600 | ns_2_bp | 104 | 21.9 | 185 | 81.8 | 0.664 | 7 | 7 | *Y. lipolytica_OD600* | 5 | 11.93 |
| *Y. lipolytica* | OD600 | ns_2 | 106 | 28.1 | 183 | 77.7 | 0.669 | 7 | 7 | *Y. lipolytica_OD600* | 9 | 14.17 |
| *Y. lipolytica* | OD600 | ns_3 | 116 | 29.8 | 201 | 85.9 | 0.568 | 7 | 7 | *Y. lipolytica_OD600* | 13 | 24.84 |
| *Y. lipolytica* | OD600 | ns_4 | 106 | 10.4 | 201 | 95.5 | 0.618 | 7 | 7 | *Y. lipolytica_OD600* | 8 | 14.17 |
| *K. marxianus* | DCW | poly_1 | 9.79 | 6.25 | 13.3 | 3.54 | 0.569 | 7 | 7 | *K. marxianus_DCW* | 14 | 71.13 |
| *K. marxianus* | DCW | poly_2 | 6.84 | 4.85 | 8.82 | 1.98 | 0.816 | 7 | 7 | *K. marxianus_DCW* | 13 | 19.56 |
| *K. marxianus* | DCW | poly_3 | 5.78 | 3.24 | 8.32 | 2.54 | 0.856 | 7 | 7 | *K. marxianus_DCW* | 2 | 1.13 |
| *K. marxianus* | DCW | poly_4 | 6.00 | 3.45 | 8.54 | 2.55 | 0.843 | 7 | 7 | *K. marxianus_DCW* | 6 | 4.89 |
| *K. marxianus* | DCW | exponential | 6.24 | 3.26 | 9.21 | 2.98 | 0.816 | 7 | 7 | *K. marxianus_DCW* | 11 | 9.06 |
| *K. marxianus* | DCW | freundlich | 6.14 | 3.85 | 8.43 | 2.29 | 0.844 | 7 | 7 | *K. marxianus_DCW* | 9 | 7.43 |
| *K. marxianus* | DCW | bs_1 | 6.07 | 4.13 | 8.02 | 1.94 | 0.854 | 7 | 7 | *K. marxianus_DCW* | 7 | 6.21 |
| *K. marxianus* | DCW | bs_2 | 5.84 | 3.24 | 8.44 | 2.60 | 0.850 | 7 | 7 | *K. marxianus_DCW* | 3 | 2.18 |
| *K. marxianus* | DCW | bs_3 | 6.08 | 3.67 | 8.49 | 2.41 | 0.844 | 7 | 7 | *K. marxianus_DCW* | 8 | 6.28 |
| *K. marxianus* | DCW | bs_4 | 5.86 | 3.81 | 7.90 | 2.05 | 0.861 | 7 | 7 | *K. marxianus_DCW* | 4 | 2.42 |
| *K. marxianus* | DCW | ns_2_bp | 6.37 | 4.29 | 8.44 | 2.08 | 0.841 | 7 | 7 | *K. marxianus_DCW* | 12 | 11.37 |
| *K. marxianus* | DCW | ns_2 | 6.17 | 4.04 | 8.29 | 2.13 | 0.850 | 7 | 7 | *K. marxianus_DCW* | 10 | 7.84 |
| *K. marxianus* | DCW | ns_3 | 5.72 | 3.07 | 8.37 | 2.65 | 0.855 | 7 | 7 | *K. marxianus_DCW* | 1 | 0.00 |
| *K. marxianus* | DCW | ns_4 | 5.99 | 3.75 | 8.22 | 2.24 | 0.854 | 7 | 7 | *K. marxianus_DCW* | 5 | 4.67 |
| *K. marxianus* | OD600 | poly_1 | 88.8 | 38.7 | 139 | 50.2 | 0.107 | 7 | 7 | *K. marxianus_OD600* | 14 | 68.43 |
| *K. marxianus* | OD600 | poly_2 | 71.0 | 34.6 | 107 | 36.4 | 0.379 | 7 | 7 | *K. marxianus_OD600* | 13 | 34.64 |
| *K. marxianus* | OD600 | poly_3 | 62.0 | 22.5 | 101 | 39.5 | 0.465 | 7 | 7 | *K. marxianus_OD600* | 5 | 17.56 |
| *K. marxianus* | OD600 | poly_4 | 64.5 | 25.8 | 103 | 38.7 | 0.417 | 7 | 7 | *K. marxianus_OD600* | 6 | 22.25 |
| *K. marxianus* | OD600 | exponential | 52.7 | 6.11 | 99.3 | 46.7 | 0.489 | 7 | 7 | *K. marxianus_OD600* | 1 | 0.00 |
| *K. marxianus* | OD600 | freundlich | 59.1 | 20.1 | 98.1 | 39.0 | 0.464 | 7 | 7 | *K. marxianus_OD600* | 3 | 12.03 |
| *K. marxianus* | OD600 | bs_1 | 64.6 | 24.2 | 105 | 40.5 | 0.464 | 7 | 7 | *K. marxianus_OD600* | 7 | 22.57 |
| *K. marxianus* | OD600 | bs_2 | 65.4 | 18.0 | 113 | 47.4 | 0.340 | 7 | 7 | *K. marxianus_OD600* | 8 | 23.96 |
| *K. marxianus* | OD600 | bs_3 | 67.2 | 37.0 | 97.4 | 30.3 | 0.452 | 7 | 7 | *K. marxianus_OD600* | 10 | 27.45 |
| *K. marxianus* | OD600 | bs_4 | 61.2 | 31.4 | 91.1 | 29.9 | 0.580 | 7 | 7 | *K. marxianus_OD600* | 4 | 16.13 |
| *K. marxianus* | OD600 | ns_2_bp | 67.7 | 30.1 | 105 | 37.6 | 0.420 | 7 | 7 | *K. marxianus_OD600* | 12 | 28.35 |
| *K. marxianus* | OD600 | ns_2 | 67.2 | 29.7 | 105 | 37.5 | 0.429 | 7 | 7 | *K. marxianus_OD600* | 9 | 27.38 |
| *K. marxianus* | OD600 | ns_3 | 56.7 | 19.2 | 94.3 | 37.6 | 0.508 | 7 | 7 | *K. marxianus_OD600* | 2 | 7.63 |
| *K. marxianus* | OD600 | ns_4 | 67.5 | 35.0 | 100 | 32.6 | 0.439 | 7 | 7 | *K. marxianus_OD600* | 11 | 28.07 |

**Key Model Classes:**

- Polynomials (poly_1–poly_4): Global curve fitting, degrees 1–4. Exponential & freundlich: Parametric nonlinear models. Basic splines (bs_1–bs_4): Piecewise polynomials with detected breakpoints. Natural splines (ns_2–ns_4): Constrained splines with automatic knot placement

# V: Residual Diagnostics and Heteroscedasticity Analysis

Comprehensive residual diagnostics for selected models, including heteroscedasticity assessment via Breusch-Pagan tests, variance ratio quantification, and visual inspection of residual patterns. This analysis confirms that while heteroscedasticity is present (physically expected due to multiple light scattering at high cell densities), the distribution-free nature of leave-one-batch-out cross-validation ensures unbiased performance estimates.

**Table S5. Heteroscedasticity Diagnostics for Selected Models**

| **Species** | **Feature** | **Model** | **N_obs** | **BP_pvalue** | **Var_Ratio** | **Severity** | **Max_Abs_Std_Res** | **N_outliers_3SD** |
| --- | --- | --- | --- | --- | --- | --- | --- | --- |
| *P. pastoris* | DCW | Natural spline 4-df | 63 | <0.001 | 7.09 | High | 2.97 | 0 |
| *P. pastoris* | OD600 | Natural spline 3-df | 63 | 0.0069 | 22.25 | High | 3.55 | 1 |
| *Y. lipolytica* | DCW | freundlich power-law | 51 | 0.2434 | 4.23 | High | 2.75 | 0 |
| *Y. lipolytica* | OD600 | Exponential | 51 | 0.6794 | 82.22 | High | 4.03 | 1 |
| *K. marxianus* | DCW | Natural spline 3-df | 59 | <0.001 | 26.33 | High | 3.13 | 1 |
| *K. marxianus* | OD600 | Exponential | 59 | <0.001 | 565.69 | High | 3.13 | 1 |

**Interpretation:**

BP_pvalue: Breusch-Pagan test p-value (p < 0.05 indicates heteroscedasticity). Var_Ratio: Ratio of variance between upper and lower tertiles of fitted values. Severity: Classification based on variance ratio (High: >3×). Max_Abs_Std_Res: Maximum absolute standardized residual. N_outliers_3SD: Number of observations with |standardized residuals| > 3

**VI: R Script for Reproducible Analysis**

Annotated R analysis pipeline implementing the full reproducibility workflow described in this manuscript: nested leave-one-batch-out cross-validation (LOBO-CV), model selection via the 5% parsimony criterion, breakpoint detection via segmented regression, cross-species ANCOVA, separated nested LOBO-CV by sensor regime (below/above breakpoint), and generation of all figures and tables reported in this manuscript and the supplementary material. Full source code, raw datasets, and complete output files are publicly available in the Zenodo repository.

**Script Information**

- File: yeastODsoft_v1.0.0.R
- Version: v1.0.0 (April 2025)
- Lines of code: ~3,900
- R version required: ≥ 4.3.0
- Primary dependencies: splines, segmented, dplyr, ggplot2, ggpubr, ggtext, ggnewscale, readxl, lmtest

**Key Sections**

Pipeline Architecture

The script is organized as a single continuous pipeline of 19 sequential sections that loads the data and reproduces every quantitative result reported in the manuscript and this Supplementary Material.

Sections 1–10 perform the core modeling: data loading and preprocessing (Section 1); definition of the 14-model bank (Section 2); breakpoint detection via segmented regression on training data only (Section 3); nested leave-one-batch-out cross-validation (Section 4); model selection by the parsimony criterion with a 5% RMSE_CV tolerance (Section 5); final model fitting to the complete dataset (Section 6); main execution and output generation (Section 7); tolerance-sensitivity analysis with breakpoint variability for Table S1 (Section 8); and the summary tables (Sections 9–10).

Sections 11–12 generate Figures 2 and 3, the experimental-data panels, and the heteroscedasticity diagnostics together with the residual-diagnostic Figures S4a–c.

Sections 13–19 reproduce the remaining inferential results cited in the manuscript: the OD₆₀₀/DCW error ratio by density bins (Section 13); the sub-range RMSE comparison below and above the breakpoint (Section 14); the curvature test below the breakpoint via local quadratic regression (Section 15); the cross-species ANCOVA for universality testing (Section 16); the quasi-linear regime comparison of Figure S2 (Section 17); the cell-morphometry and Mie size-parameter analysis generating Tables 4 and 5 (Section 18); and the partitioned below/above-breakpoint performance for Table S1B (Section 19).

Together, the pipeline generates manuscript Tables 2, 3, 4, 5 and 6 and Figures 2 and 3, and supplementary Tables S1, S1B, S2, S3, S4 and S5 and Figures S2 and S4a–c.

**Output Files Generated**

**Tables (CSV format):**

- TABLE_S4_model_performance.csv — Complete LOBO-CV ranking of the 14 candidate models across the six yeast–feature combinations (84 rows total; supplementary Table S4).
- TABLE2_final_models.csv — Selected software and calculator models with breakpoints (manuscript Table 2).
- TABLE6_predictions_canonical_OD.csv — Canonical model predictions at reference OD₈₆₀ values (manuscript Table 6).
- TABLE3_calculator_equations.csv — Closed-form calculator equations for the selected models (manuscript Table 3).
- TABLE_S1_breakpoint_summary.csv — Breakpoint mean, SD, and CV across LOBO-CV folds (Table S1).
- TABLE_S1B_partitioned_performance.csv — Partitioned out-of-fold performance below and above the breakpoint for the selected model, re-fit locally in each partition by LOBO-CV (Table S1B).
- TABLE_S2_tolerance_sensitivity.csv — Model selection at parsimony tolerance thresholds 2.5%, 5%, 7.5%, and 10% (Table S2).
- TABLE_S3_baseline.csv — Linear (poly_1) baseline performance metrics (Table S3).
- TABLE_S5_heteroscedasticity.csv — Breusch-Pagan tests, variance ratios, and outlier counts for selected models (Table S5). residuals_all.csv — Per-observation out-of-fold residuals for all models (used internally by Section 12 and Figures S4a–c). results_complete.rds — Complete R results object containing all fitted models, breakpoints, and CV metrics (binary; for advanced re-analysis).

**Figures (PDF and PNG format):**

- FIG2_complete_panel.png — Six-panel scatterplot of OD₈₆₀ versus DCW and OD₆₀₀ for the three species (manuscript Figure 2).
- FIG3_complete_panel.png — Six-panel display of top-ranked regression models with species-specific breakpoints (manuscript Figure 3); also generated as six individual panels FIG3a–f.
- FIGURE_S4a_Pichia_pastoris.pdf/png — Residual diagnostics for P. pastoris (residuals vs. fitted and scale-location plots for both DCW and OD₆₀₀ models).
- FIGURE_S4b_Yarrowia_lipolytica.pdf/png — Residual diagnostics for Y. lipolytica.
- FIGURE_S4c_Kluyveromyces_marxianus.pdf/png — Residual diagnostics for K. marxianus.
- FIGURE_S2_quasi_linear_comparison.pdf/png — Quasi-linear regime comparison across species (Figure S2).

**Usage Instructions**

**To reproduce all analyses and figures:**

i) place Raw_data.xlsx and datos_samples_microscopio_v1.0.xlsx in the same directory as the script;

(ii) install required R packages with install.packages(c('splines', 'segmented', 'dplyr', 'ggplot2', 'ggpubr', 'ggtext', 'ggnewscale', 'readxl', 'lmtest'));

(iii) execute the script with source('yeastODsoft_v1.0.0.R'). All output files are written to the outputs_v1.0.0/ subdirectory. Total execution time is approximately 3–5 minutes on standard hardware. The full source code, raw datasets, and complete output files are publicly available in the Zenodo repository
